# RNAdetector: a free user-friendly stand-alone and cloud-based system for RNA-Seq data analysis

**DOI:** 10.1186/s12859-021-04211-7

**Published:** 2021-06-03

**Authors:** Alessandro La Ferlita, Salvatore Alaimo, Sebastiano Di Bella, Emanuele Martorana, Georgios I. Laliotis, Francesco Bertoni, Luciano Cascione, Philip N. Tsichlis, Alfredo Ferro, Roberta Bosotti, Alfredo Pulvirenti

**Affiliations:** 1 Department of Clinical and Experimental Medicine, Bioinformatics Unit, University of Catania, Catania, Italy; 2 Department of Cancer Biology and Genetics, The Ohio State University, Columbus, OH USA; 3 Department of Physics and Astronomy, University of Catania, Catania, Italy; 4 Nerviano Medical Sciences, Nerviano, Milan, Italy; 5 Regional Referral Centre for Rare Lung Diseases, A. O. U. “Policlinico-Vittorio Emanuele”, Department of Clinical and Experimental Medicine, University of Catania, Catania, Italy; 6 Institute of Oncology Research, Bellinzona, Switzerland

**Keywords:** RNA-seq, Stand-alone software, Cloud deployment, Pipeline, Docker, ncRNAs, Differential expression analysis, Pathway analysis

## Abstract

**Background:**

RNA-Seq is a well-established technology extensively used for transcriptome profiling, allowing the analysis of coding and non-coding RNA molecules. However, this technology produces a vast amount of data requiring sophisticated computational approaches for their analysis than other traditional technologies such as Real-Time PCR or microarrays, strongly discouraging non-expert users. For this reason, dozens of pipelines have been deployed for the analysis of RNA-Seq data. Although interesting, these present several limitations and their usage require a technical background, which may be uncommon in small research laboratories. Therefore, the application of these technologies in such contexts is still limited and causes a clear bottleneck in knowledge advancement.

**Results:**

Motivated by these considerations, we have developed RNAdetector, a new free cross-platform and user-friendly RNA-Seq data analysis software that can be used locally or in cloud environments through an easy-to-use Graphical User Interface allowing the analysis of coding and non-coding RNAs from RNA-Seq datasets of any sequenced biological species.

**Conclusions:**

RNAdetector is a new software that fills an essential gap between the needs of biomedical and research labs to process RNA-Seq data and their common lack of technical background in performing such analysis, which usually relies on outsourcing such steps to third party bioinformatics facilities or using expensive commercial software.

**Supplementary Information:**

The online version contains supplementary material available at 10.1186/s12859-021-04211-7.

## Background

Next-Generation Sequencing (NGS) technologies are boosting our understanding of the molecular mechanisms underlying prokaryotic and eukaryotic cell signaling, development, and organization [1]. These technologies allow the sequencing of entire genomes in a few days, yielding the possibility to detect gene mutations or polymorphisms (e.g., CNV, SNPs, INDEL, STR) potentially associated with different diseases [1]. NGS is also extensively used for transcriptome profiling (RNA-Seq), allowing identifying differentially expressed genes, splicing variants, or complex gene rearrangements that could represent driver events in specific diseases [2].

Moreover, RNA-Seq can also be used to detect non-coding RNAs (ncRNAs), namely, RNA molecules that do not encode for proteins but represent a considerable amount of the transcriptome involved in many aspects of cell physiology [2, 3]. Indeed, they act by regulating a broad spectrum of cellular processes, controlling gene expression, and contributing to genome organization and stability [3]. Upon the increasing research interest in ncRNAs, identifying the different subclasses has emerged as a critical issue. Indeed, RNA-Seq produces a dramatically higher amount of data than other traditional technologies, such as Real-Time PCR or microarray, demanding fast and effective computational approaches [4].

For this purpose, several pipelines have been developed for the analysis of gene expression from RNA-Seq data. Relevant examples include: ArrayExpressHTS (https://www.bioconductor.org/packages/release/bioc/html/ArrayExpressHTS.html), BioJupies [5], BioWardrobe [6], DEWE [7], easyRNASeq [8], ExpressionPlot [9], FX [10], GENE-counter [11], GeneProf [12], Grape RNA-Seq [13], MAP-RSeq [14], NGScloud [15, 16], RAP [17], RobiNA [18], RSEQREP [19], RSEQtools [20], RseqFlow [21], S-MART [22], TCW [23], TRAPLINE [24] and wapRNA [25]. In addition, other pipelines have been developed for the analysis of different ncRNA classes: DSAP [26], miRanalyzer [27], miRExpress [28], miRNAkey [29], iMir [30], CAP-miRSeq [31], mirTools 2.0 [32], sRNAtoolbox [33], miRDeep 2 [34], and MapMi [35] for microRNAs (miRNAs); piPipes [36], PILFER [37], piRNAPredictor [38] and PIANO [39] for piwi-associated RNAs (piRNAs); and UClncR [40] for long non-coding RNAs (lncRNAs).

More recent pipelines have been released to analyze small RNA-Seq data allowing the analysis of more than one ncRNAs class such as iSmaRT [41], iSRAP [42], miARma-Seq [43], Oasis 2 [44], SPORTS1.0 [45], sRNAnalyzer [46], and sRNApipe [47]. However, some of these tools present several limitations and shortcomings which have negatively impacted their usage by non-expert users: (1) no Graphical User Interface but only command-line shell; (2) software dependencies before the pipeline installation; (3) support only for UNIX operating systems; (4) static workflow (they do not allow to choose the tool to be used in each step of the pipeline); (5) not suitable for the analysis of the whole transcriptome (e.g., mRNAs and\or few ncRNA classes supported); (6) no downstream analysis modules (i.e., differential expression analysis or pathway analysis); (7) only a few species supported.

To analyze the state of the art, in a recent review, we tested some novel RNA-Seq pipelines highlighting the need for more comprehensive, flexible, and easy-to-use free tools that could be used either for research or biomedical purposes [48]. In particular, within a biomedical research setting, the availability of stand-alone offline software is crucial to guarantee the data safety of human/patient-derived RNA-Seq data. To include researchers with no prior knowledge of computer programming, we introduce *RNAdetector,* a free cross-platform, and user-friendly RNA-Seq data analysis software which can be used locally or in cloud environments by mean of an easy-to-use Graphical User Interface (GUI) allowing the analysis of coding and ncRNAs from RNA-Seq datasets of any sequenced biological species.

## Implementation

### Software implementation

*RNAdetector* is a client–server application developed to simplify deployment and usage. The server has been developed in PHP, Bash, and R. All server code and dependencies are deployed through a Docker container for easy installation. Communication between client and server is based on an HTTP REST API specifically developed for *RNAdetector*. An internal Mysql database is used to store all server data. Authentication, API Security, and the data abstraction layer have been provided by the Laravel framework (https://laravel.com/). The Graphical User Interface (GUI) has been developed in Javascript using the Electron framework (https://electronjs.org/). Electron is an open-source framework developed and maintained by GitHub, allowing the development of desktop GUI applications using web technologies.

*RNAdetector* can be used entirely offline installed as a stand-alone desktop application on many operating systems, such as Windows Professional, macOS, and Linux. Furthermore*,* it can also be installed in servers and remotely controlled by a local installation of our app. Deployment on remote servers can be performed through docker-compose on a single machine or Kubernetes for a clustered environment. Therefore, RNAdetector can also be installed on several cloud providers such as Google Cloud Platform, Microsoft Azure, or Amazon AWS.

*RNAdetector* can perform quantification, normalization, and differential expression analysis of human, mouse, and *C.elegans* mRNAs and several classes of ncRNAs such as miRNAs, piRNAs [only for human at this moment], small nucleolar RNAs (snoRNAs), lncRNAs, transcribed ultraconserved regions (t-UCRs) [only for human at this moment], circular RNAs (circRNAs), and tRNA-derived ncRNAs. However, additional ncRNA classes can also be analyzed by uploading their genomic coordinates (in GTF or BED format) following the step-by-step procedure detailed in the user interface. To visualize the depth of coverage of mapped reads, we integrated an offline interactive genome browser based on JBrowse 2 [49]. Finally, topological pathway analysis of protein-coding genes and miRNAs can also be performed. Details about the pipeline design are described in the next section.

*RNAdetector* comes with a repository containing pre-built genomes and annotations for human, mouse, and *C.elegans.* However, other sequenced species can be analyzed by providing their FASTA genomes or transcriptomes and GTF annotations. RNAdetector can index such genomes/transcriptomes on any available algorithm such as BWA [50], Salmon [51], HISAT2 [52, 53], and STAR [54]. The user will be guided through a graphical procedure, avoiding the use of any command-line tool.

*RNAdetector* is freely available for download at https://rnadetector.atlas.dmi.unict.it/download.html. Source code and issue reporting are available at https://github.com/alessandrolaferlita/RNAdetector.

### Pipeline design

*RNAdetector* allows users to start the analysis from different input files such as FASTQ, BAM, or SAM files. We employ Trim Galore (http://www.bioinformatics.babraham.ac.uk/projects/trim_galore/) for quality trimming and adapters removal on FASTQ files. According to the input file type, the alignment strategy, and the sequencing strategy (mRNAs, small RNAs, etc.), the proper pipeline is run. For mRNAs, small ncRNAs, and lncRNAs, the alignment can be executed on a reference genome by using HISAT2 [53] or STAR [54]. It can also be executed on a reference transcriptome by using SALMON [51]. On the other hand, for circRNA analysis, reads are first mapped on the reference genome with BWA [50]. Next, they can be quantified (for circRNAs already annotated on circBase [55]), or de-novo identified and quantified by mean of CIRI 2 [56, 57] or CIRIquant [58].

*RNAdetector* stores in its remote repository human, mouse, and *C.elegans* indexed genomes and transcriptomes together with their GTF and FASTA files which can be downloaded directly from our repository through the user interface. Concerning genome-based alignment, human (HG19 and HG38), mouse (mm9 and mm10), and *C.elegans* (ce11) genomes have been indexed by using HISAT2 [52, 53], STAR [54], and BWA [50] and included in *RNAdetector* (they are present in our remote repository ready for the download). Genome annotation for human, mouse, and *C.elegans* is also allowed through custom GTF files. Specifically, we included (1) GTF files with the genomic coordinates of protein-coding genes, snoRNAs, and lncRNAs retrieved from GENCODE for human and mouse (HG19 v19, HG38 v33, mm9 vM1, mm10 vM26) and ENSEMBL (ce11 WBcel235) for *C.elegans* (2) custom GTF files with the genomic coordinates of miRNAs (retrieved from miRBase [59]), piRNAs (retrieved from piRBase [60]), and tRNA-derived ncRNAs (retrieved from tRFexplorer [61] for human and from tRFdb [62] for mouse and *C.elegans*) (3) GTF files with the genomic coordinates of human, mouse and *C.elegans* circRNAs retrieved from circBase [55] (4) and a GTF file with the genomic coordinates of human t-UCRs retrieved from UCbase [63]. Concerning transcriptome-based alignment, *RNAdetector* has custom human, mouse, and *C.elegans* transcriptomes indexed by SALMON [51], which were built by retrieving the mRNAs and lncRNAs FASTA sequences from GENCODE for human and mouse (HG19 v19, HG38 v33, mm9 vM1, mm10 vM26) and ENSEMBL (ce11 WBcel235) for *C.elegans*.

In the next two steps, reads are aligned with a reference genome or transcriptome and quantified to infer mRNAs or ncRNAs expression levels. For this purpose, *RNAdetector* allows users to select several tools and options to perform the alignment and read quantification steps. Specifically, if users choose the genome-based alignment, STAR [54] and HISAT 2 [52, 53] are the available aligners. Subsequently, read quantification can be executed by HTseq [64], FeatureCounts [65], or SALMON [51] (alignment-based mode). Instead, if users choose the transcriptome-based alignment strategy, reads are aligned and quantified by SALMON [51] in a single step for a faster and RAM saving analysis.

Once the read quantification step is performed, RNAdetector’s workflow allows performing differential expression analysis on mRNAs or ncRNAs. For this purpose, we included three of the most common tools for differential expression analysis, such as DESeq2 [66], edgeR [67], and LIMMA [68]. These three methods use different assumptions, normalization methods, and statistics to identify differentially expressed genes. Therefore, they can yield different results from the same datasets. However, we included these three methods to allow users to choose the most suitable tool for their analysis. Also, the users can perform a more rigorous analysis by combining these three methods in a meta-analysis that should highlight the more robust differentially expressed genes. Finally, miRNA-sensitive topological pathway analysis can be performed by MITHrIL [69] using the LogFC values of mRNAs and\or miRNAs obtained after the differential expression analysis step. A final report based on metaseqR [70] with a summary, tables, and figures is provided together with an additional report developed to visualize pathway analysis results. An offline genome browser based on JBrowse 2 [49] is also available to visualize the depth of coverage of mapped reads.

### Case study analysis

We selected a small RNA-Seq project publicly available on NCBI SRA (SRP183064). The analysis was performed by using *RNAdetector* and selecting the following parameters and tools from its user interface. A video of the analysis is available as Additional file 1. We started the analysis from the FASTQ files, raw reads were trimmed, and adapters were removed by selecting Trim Galore from the user interface. Trimmed reads were then aligned to the reference human genome (HG38) by selecting HISAT 2 [53] and counted by featureCounts [65]. Before the statistical testing procedure, the read counts were filtered for possible artifacts that could affect the subsequent statistical testing procedures. After that, the count table was normalized for inherent systematic or experimental biases selecting edgeR [67] as a normalization method after removing features that had zero counts over all the RNA-Seq samples. The normalized count matrix was then used for the differential expression analysis by selecting limma [68] and edgeR [67] from the RNAdetector’s user interface. Finally, to combine the statistical significance from multiple algorithms and perform a meta-analysis, the Simes correction and combination method was applied. The pathway analysis was performed by selecting the MITHrIL algorithm [69], which used the LogFC values of miRNAs obtained from the differential expression analysis step for its analysis. Pathways with FDR or adjusted p-values < 0.01 were considered impacted.

## Results

### Software introduction

*RNAdetector* was designed as an easy-to-use, flexible, cross-platform, and comprehensive pipeline, allowing users to analyze mRNAs and ncRNAs. Precisely, several classes of Human, Mouse, and *C.elegans* ncRNAs such as miRNAs, piRNAs [only for human at this moment], snoRNAs, lncRNAs, t-UCR [only for human at this moment], circRNAs, and tRNA-derived ncRNAs classes reported in tRFexplorer [61] and tRFdb [62] are already stored in the remote repository of *RNAdetector. They* can be downloaded directly through the user interface, allowing a more accessible analysis. However, any additional species whose genomes have been sequenced can also be analyzed by uploading their genomes or transcriptomes (in FASTA format) and the genomic annotations (in GTF or BED format). Specifically, *RNAdetector* allows not only the identification and quantification of the classes mentioned above, but it also provides downstream analysis modules such as differential expression analysis and miRNA-sensitive topological pathway analysis [69], allowing users to infer crucial biological information from their RNA-Seq data.

### Deployment and installation

*RNAdetector* is distributed as a Docker container and automatically installed after its first execution to manage the dependencies. No previous dependencies are needed to be installed in users’ machines, and it can be used as a simple offline desktop application with several operating systems such as Windows, macOS, and Linux. Users have only to install Docker in their machine (Docker can be installed through a user-friendly installer for Windows, Linux, and macOS) and download one of the available RNAdetector’s installers specific for his operating system. Moreover, *RNAdetector* can be installed in servers, and it can be remotely controlled by installing our front-end locally. No internet connection is needed to perform the analysis for a local setup. *RNAdetector can be used* as entirely offline stand-alone software to handle sensitive or patient-derived RNA-Seq data covered by privacy, not to be analyzed using other web-based pipelines. A summary of its system requirements is shown in Table 1.

**Table 1:** System requirements

Feature	Description
Supported operating systems	Windows Professional; macOS; Linux
Dependencies	Docker
Connectivity	Offline (For the stand-alone version internet connection is only required for the installation and updates) Online (for the cloud-based version)
Minimum System Requirements (Stand-alone version)	Processor: 6 cores processor RAM: 16 GB Hard drive: 1 Tb (space is required to store the analysis of multiple samples)
Recommended System Requirements (Stand-alone version)	Processor: 8 cores processor or greater RAM: 32 GB or more Hard drive: 2 Tb or more (space is required to store the analysis of multiple samples)

However, since *RNAdetector* leverages the power of a containerized deployment, it can be easily installed in public cloud environments, such as Google Cloud Platform, Microsoft Azure, or Amazon AWS, or local clusters through Kubernetes.

RNAdetector is freely available for download at https://rnadetector.atlas.dmi.unict.it/download.html. More details about the system requirements and setup can be found at the following link https://github.com/alessandrolaferlita/RNAdetector/wiki/Requirements-and-Setup.

### Functionalities

One of the different strengths of *RNAdetector* is its interactive and easy-to-use GUI. Our GUI has been implemented to be used by users with no computer programming background to promote its use both in small research and biomedical laboratories. Users can select several options to perform the most suitable analysis for their data through the user interface. They can select the input type (e.g., FASTQ, SAM, or BAM), and per the RNA-Seq strategy, the class of RNAs they want to analyze, such as mRNAs, small ncRNAs (miRNAs, snoRNAs, piRNAs, tRNA-derived ncRNAs), lncRNA, t-UCR, or circRNAs. To give extreme flexibility to our software, users can also select which tool they want to use for each step of the pipeline and their parameters (for expert users, custom parameters can also be provided).

For the alignment, users can choose HISAT2 [53] or STAR [54] for alignment against a reference genome or SALMON [51] for quantification on a reference transcriptome. The alignment strategy is a critical point for RNA-Seq data analysis, and it must be evaluated accordingly with the purpose of the analysis. For example, the alignment of reads to a reference transcriptome with SALMON is the suggested strategy to analyze the expression profile of splicing-variant transcripts. On the other hand, for other RNA molecules that are not subject to alternative-splicing, such as small ncRNAs, or to summarize the transcript expression at gene-level, the alignment on a reference genome is the default option. Moreover, to see the depth of coverage of the mapped reads produced during the analysis along the entire genome, an offline interactive genome browser based on JBrowse 2 [49] was integrated into the user interface. Concerning read counting, it can also be performed by choosing one of the several available tools such as HTseq [64], FeatureCount [65], or SALMON [51].

However, for circRNAs, the pipeline has a strict workflow that consists of aligning the reads on the reference genome with BWA [50], and then *de-novo* or annotated-based identification and quantification by using CIRI 2 [56, 57] or CIRIquant [58].

Optional downstream analysis modules on the identified and quantified mRNAs and ncRNAs are also available. Specifically, *RNAdetector* allows users to perform differential expression analysis and miRNA-sensitive topological pathway analysis. Normalization and differential expression analysis can be performed by DESeq2 [66], edgeR [67], LIMMA [68], or by the combination of these three methods. miRNA-sensitive topological pathway analysis is executed by the MITHrIL algorithm [69]. MITHrIL fully exploits the topological information encoded by pathways when computing perturbation scores. Pathways are modeled as complex graphs where each node is a biological element (protein-coding gene, miRNA, or metabolite), and each edge is an interaction between them. Even though thousands of genes are not annotated in pathways, and existing annotations may be inaccurate, graphs in these databases provide a more detailed view of biological processes within the cell, helping interpret high-throughput experiments [71].

**Fig. 1:**
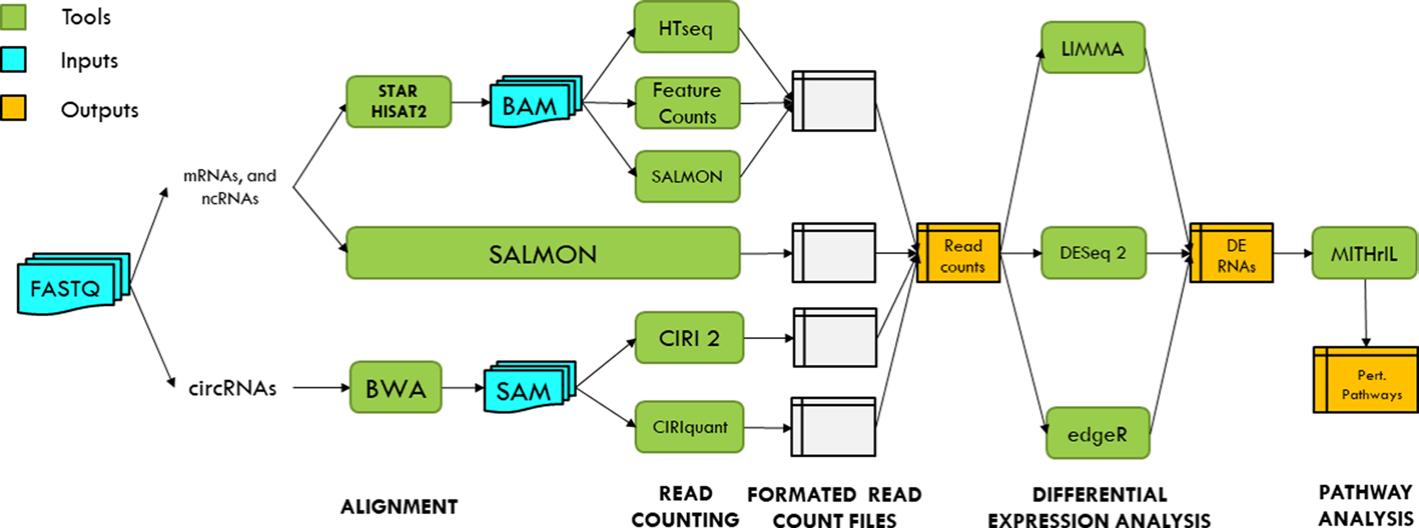
RNAdetector’s pipeline. This figure shows a schematic representation of RNAdetector’s pipeline with its tools, input, and output files

All the tools available in *RNAdetector* are well-known and widely used freeware tools with tested and proven efficiency individually used by bioinformaticians to analyze RNA-Seq data and integrated into *RNAdetector* to simplify users’ experience. A schematic picture of the pipeline’s workflow is reported in Fig. 1.

Finally, although the *RNAdetector* repository contains genomes and annotations for human, mouse, and *C.elegans* RNA-Seq data analysis, it can also be used with any other sequenced organism by providing the reference genome or transcriptome and the genomic annotations of the RNA molecules to be analyzed.

A summary of RNAdetector’s functionalities is shown in Table 2, together with supported species, RNA types, and input and output files.

**Table 2:** RNAdetector’s supported analysis, species, RNA types and files

Feature	Description
Input Files	FASTQ; BAM; SAM
Supported Analysis	Quantification; Differential expression analysis; Pathway analysis
Supported Species	Human; Mouse; *C. elegans.* Additional sequenced species can be analyzed by uploading their genome and\or transcriptome in FASTA format following the step-by-step procedure detailed in the user interface
Supported RNA types	mRNAs; miRNAs; snRNAs; snoRNAs; piRNA [only for human at this moment]; tsRNAs; tUCR [only for human at this moment]; lncRNAs; circRNAs. Additional ncRNAs classes can be analyzed by uploading their genomic coordinates in GTF or BED format following the step-by-step procedure detailed in the user interface
Output Files	Graphical final report for both Differential Expression Analysis and Pathway Analysis with summary of the results, figures, and tables. Text files with raw counts, normalized counts, differentially expressed gene and perturbated pathway can also be downloaded

A complete user’s guide is available at https://github.com/alessandrolaferlita/RNAdetector/wiki.

### Final report

To guarantee a straightforward interpretation of the results, we believed that an interactive and exhaustive report with a summary of the results, tables, and several plots is crucial. Specifically, we developed two reports for the differential expression and pathway analysis modules, respectively. The report for the differential expression analysis is based on a modified metaseqR [70] package. Precisely, it shows a summary of the results with all the parameters and input options used for the analysis, and several figures to show the quality of the sequencing and its results (Multidimensional scaling, RNA-Seq reads noise, Correlation plots, Pairwise scatterplots, Box Plots, RNA composition plots, Gene/transcript length bias plots, Mean-difference plots, Mean–variance plots, Volcano plots, DEG heatmaps, and Meta-analysis Venn diagrams). The final report contains high-quality publication-ready pictures generated by *RNAdetector* for easy results interpretation. Besides, an interactive table for each comparison is also present. Finally, the entire report for the differential expression analysis can be downloaded as a self-contained ZIP archive or viewed directly through the user interface. Like the differential expression analysis report, the pathway analysis report summarizes the results and several interactive figures and tables that show the biological pathways that have been found impacted. In this case, the entire report can be downloaded as a self-contained ZIP archive or viewed directly through the user interface. In addition to the final reports, users can also download all figures shown in the final reports and text files with raw or normalized read count matrices, differentially expressed mRNAs or ncRNAs, and impacted pathways.

### Case study

**Fig. 2:**
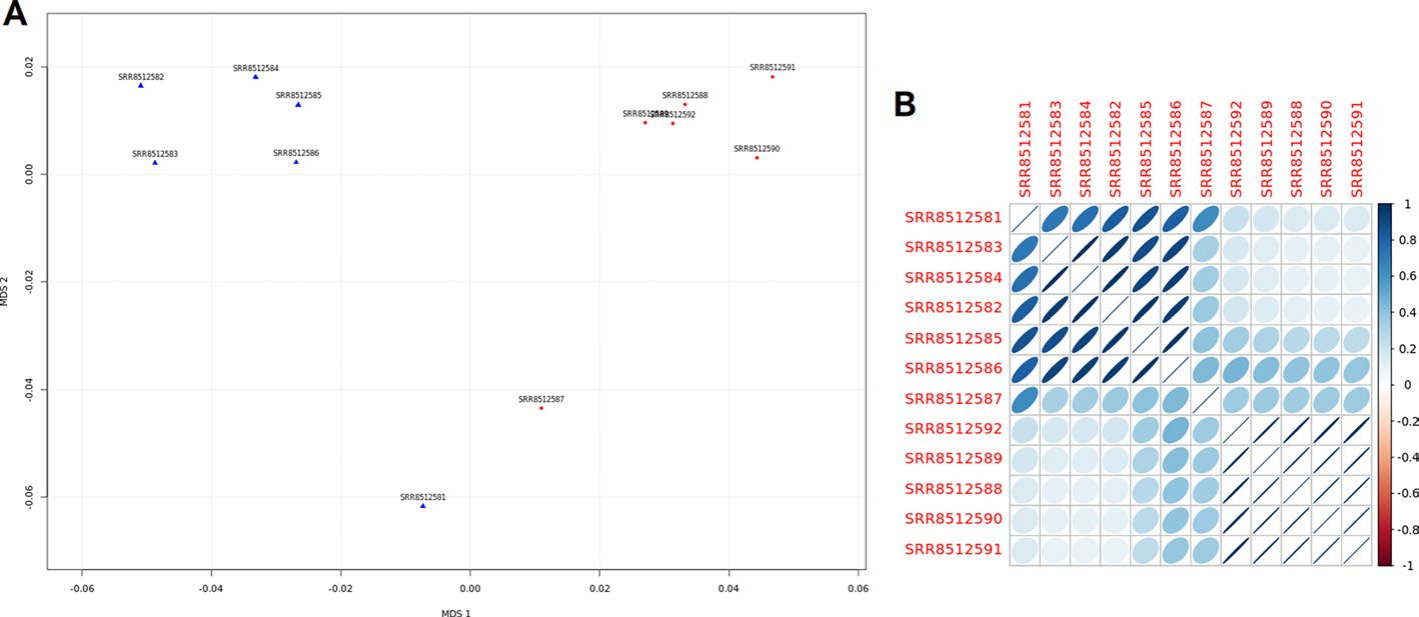
The Multi-Dimensional Scaling (MDS) plot and Correlogram’ plot. (**A**)This figure shows the MDS plot generated by RNAdetector in its final report. CRC samples are indicated with blue triangles while the adjacent normal tissue samples are indicated as red circles. MDS allows to perform quality control analysis and it can be interpreted as follows: when the distance among samples of the same biological condition in the MDS space is small, this is an indication of high correlation among them. A larger distance indicates a low correlation and reproducibility among samples. In this case, with the exception of two samples, CRC and normal tissue samples form two distinct clusters (samples are named with their SRR identifiers). (**B**) This figure shows a ‘correlogram’ plot generated by RNAdetector in its final report. Samples are hierarchically clustered and the correlations between samples are presented as ellipses inside each cell. Each cell represents a pairwise comparison and each correlation coefficient is represented by an ellipse whose ‘diameter’, direction, and color depict the accordance for that pair of samples. Highly correlated samples are depicted as ellipses with narrow diameters, while poorly correlated samples are depicted as ellipses with wide diameters. From the correlogram plot is evident how CRC and normal tissue samples form two distinct groups (samples are named with their SRR identifiers)

**Fig. 3:**
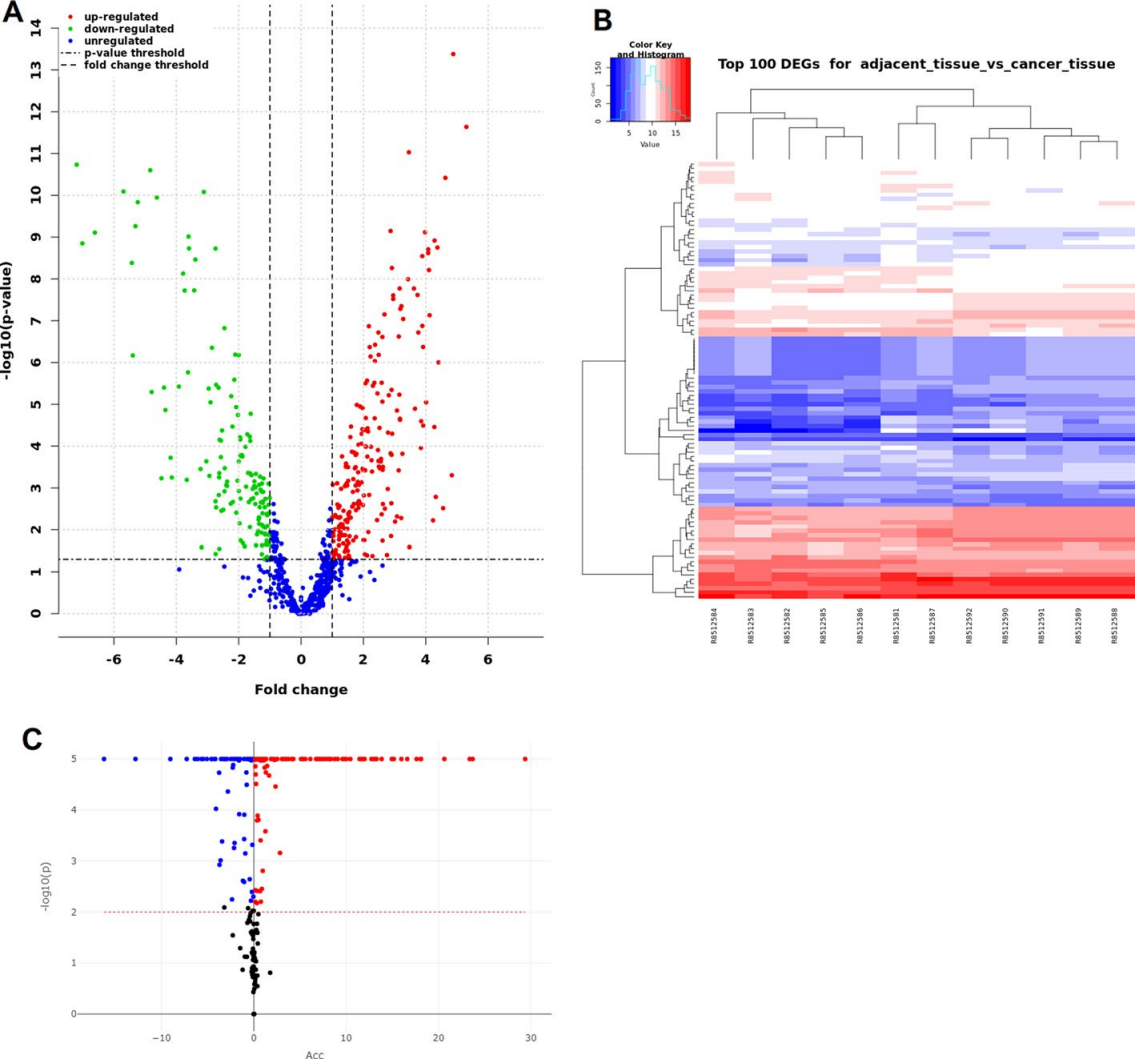
Volcano plots and heatmap of dysregulated small ncRNAs and impacted pathways. (**A**) This figure shows a volcano plot generated by RNAdetector in its final report with the up-regulated (red) and down-regulated (green) small ncRNAs identified after the comparison between CRC samples vs adjacent normal tissue samples. A volcano plot combines the results of a statistical test with the magnitude of the change enabling quick visual identification of those genes that display large-magnitude changes that are also statistically significant. The horizontal dashed line indicates the threshold for statistical significance, while the vertical dashed lines are the thresholds for biological significance. (**B**) This figure shows a heatmap generated by RNAdetector with the top 100 differentially expressed small ncRNAs. The top 100 deregulated small ncRNAs were selected for their statistical significance in terms of smaller adjusted p-value. Also with the top 100 deregulated small ncRNAs, CRC and normal tissue samples form two distinct clusters (samples are named with their SRR identifiers). (**C**) This figure shows a volcano plot generated by RNAdetector in its pathway analysis report with the significantly impacted pathways. All significantly impacted pathways are represented in terms of their measured accumulation (x-axis) and the significance (y-axis). The dotted lines represent the thresholds used to select significantly impacted pathways. Significantly impacted pathways with positive accumulation are shown in red, while the negative ones in blue

To clearly show how easily a complete analysis with *RNAdetector* can be performed, we chose a public small RNA-Seq project available on NCBI SRA (SRP183064). We performed an analysis identifying the differentially expressed small ncRNAs and the impacted biological pathways. A short video tutorial showing all the steps of the analysis is available as Additional file 1. More precisely, we used very recent small RNA-Seq datasets of Colon Rectal Cancer (CRC) [72], and we compared the expression profiles of the CRC samples against the adjacent normal tissue samples of the same patients. The goal was to identify the differentially expressed miRNAs, snoRNAs, and tRNA-derived ncRNAs and the impacted biological pathways. The total number of samples was 12 (6 CRC samples and 6 adjacent normal tissue samples). Before starting the differential expression analysis, *RNAdetector* performs some quality control analyses whose results are included in the final report. For example, through a Multi-Dimensional Scaling (MDS) analysis, it is evident that (except for two samples) the CRC samples and the normal adjacent tissue samples identify two distinct clusters (Fig. 2A). Also, the excellent quality of the samples was confirmed through a correlation analysis (Fig. 2B). *RNAdetector* identified 426 differentially expressed small ncRNAs (*p* value 0.05) through the differential expression analysis, 357 out of 426 with an FDR or adjusted *p* value < 0.05. More Precisely, a tRNA-fragment 3’ (tRF-3) named tRFdb-3033a, a tsRNAs named ts-112, 87 snoRNAs, and 337 miRNAs were found differentially expressed. The complete list of the differentially expressed small ncRNA can be found in the Additional file 2, while in Fig. 3A, they are displayed in a volcano plot generated by *RNAdetector* in its final report. The numbers mentioned above refer to the combined analysis performed by LIMMA and edgeR, selecting only the small ncRNAs found differentially expressed by both approaches. A heatmap generated by *RNAdetector* with the top 100 differentially expressed small ncRNAs is also shown in Fig. 3B, confirming the presence of two distinct clusters. After the differential expression analysis, the deregulated miRNAs were used for the pathway analysis. *RNAdetector* allows performing miRNA-sensitive topological pathway analyses by using the MITHrIL algorithm [69]. In this experiment, 166 pathways were found significantly impacted (FDR or adjusted p-value threshold of 0.01) in the CRC samples compared with adjacent normal tissue samples due to the alteration in miRNAs’ expression profiles. The complete list of the impacted pathways can be found in the Additional file 3, while in Fig. 3C, we show a volcano plot generated by *RNAdetector* in its final pathway analysis report.

### Feature comparison of RNAdetector against previous pipelines

To highlight the extensive feature’ set of *RNAdetector,* we compared our tool against 19 pipelines for RNA-Seq data analysis and seven pipelines for ncRNA-Seq analysis.

Among the RNA-Seq analysis pipelines, we selected ArrayExpressHTS (https://www.bioconductor.org/packages/release/bioc/html/ArrayExpressHTS.html), BioJupies [5], BioWardrobe [6], DEWE [7], easyRNASeq [8], ExpressionPlot [9], FX [10], GENE-counter [11], GeneProf [12], Grape RNA-Seq [13], MAP-RSeq [14], NGScloud [15, 16], RAP [17], RobiNA [18], RSEQREP [19], RSEQtools [20], RseqFlow [21], S-MART [22], TCW [23], TRAPLINE [24] and wapRNA [25]. Although interesting, some of them present shortcomings that may have negatively impacted their usage among non-expert users (a table that shows the features of *RNAdetector* compared with the other methods is presented in the Additional file 4). For instance, except for web-based and cloud-based pipelines that do not require a local installation (e.g., BioJupies [5], FX [10], GeneProf [12], NGScloud [15, 16], RAP [17], RSEQREP [19], TRAPLINE [24], and wapRNA [25]), all of them have dependencies that have to be previously installed in the user’s computer, or they require the installation and setup of virtual machines. In addition, some of these pipelines do not have GUIs (e.g. ArrayExpressHTS, easyRNASeq [8], GENE-counter [11], Grape RNA-Seq [13], MAP-RSeq [14], RSEQREP [19], RSEQtools [20], and RseqFlow [21]). This shortcoming limits their usage by users who are not confident with the command-line shell. Another limiting aspect of such pipelines is their low flexibility. Some of these pipelines have no customizable work-flows (e.g., BioJupies [5], BioWardrobe [6], ExpressionPlot [9], FX [10], Grape RNA-Seq [13], MAP-RSeq [14], RobiNA [18], RSEQREP [19], RseqFlow [21], S-MART [22], TCW [23], TRAPLINE [24], and wapRNA [25]) and, therefore, they do not allow users to select the proper tools and options in each step of the pipeline (e.g., alignment, read quantification, differential expression analysis, etc.). Finally, important features of an RNA-Seq analysis pipeline include (1) downstream analysis modules, (2) graphical and interactive final report for an easy interpretation of the results, and (3) the availability of ncRNA analysis settings. Concerning the downstream analysis modules, ArrayExpressHTS, easyRNASeq [8], Grape RNA-Seq [13], RSEQtools [20] do not present any downstream analysis module. On the contrary, BioWardrobe [6], ExpressionPlot [9], RobiNA [18], and S-MART [22] include at least one tool for the differential expression analysis module while BioJupies [5], DEWE [7], GENE-counter [11], GeneProf [12], NGScloud [15, 16] RAP [17], RSEQREP [19], RseqFlow [21], TCW [23], TRAPLINE [24], and wapRNA [25] allow to perform differential expression analysis and other different downstream analyses (see Additional file 4 for further details). Other pipelines do not generate any interactive graphical final report with a summary of the results together with figures and tables (e.g., ArrayExpressHTS, easyRNASeq [8], FX [10], GENE-counter [11], NGScloud [15, 16], RSEQtools [20], RseqFlow [21], and TRAPLINE [24]) making more difficult the interpretation of the obtained results. Finally, as an extremely limiting aspect, none of these pipelines allows specific settings for ncRNA analyses. Only TRAPLINE [24] and wapRNA [25] enable the analysis of miRNAs and their targets. Lastly, some of these pipelines such as BioWardrobe [6], DEWE [7], ExpressionPlot [9], FX [10], GeneProf [12], RseqFlow [21], and wapRNA [25] are no longer maintained. *RNAdetector* overcomes all these limitations by including all these features mentioned above, which might be individually present in specific pipelines, with new additional ones in a single integrated solution to simplify the user’s experience.

We also compared the features of *RNAdetector* against some recent ncRNA pipelines, which can analyze more than one class of ncRNAs from RNA-Seq data. These pipelines are iSmaRT [41], iSRAP [42], miARma-Seq [43], Oasis 2 [44], SPORTS1.0 [45], sRNAnalyzer [46], and sRNApipe [47]. All these pipelines can identify and quantify different sets of ncRNAs classes with variable accuracy [48]. However, many of them present similar limitations to those of the previously discussed RNA-Seq pipelines (further details of these feature comparisons are reported in the Additional file 5). All but miARma-Seq [43] (that is deployed by docker container), Oasis 2 [44] (that is a web-based application), and sRNApipe [47] (that is a Galaxy server application) are standalone tools that need several dependencies to be previously installed on users’ machines. Moreover, only iSmaRT [41], Oasis 2 [44], and sRNApipe [47] have a GUI (for the last two is web interface). None of them generate a final graphical report with a summary of the results and figures to help users interpret the results. However, all but sRNAnalyzer [46] generate text files containing the analysis results and several plots. Also, for such pipelines, users have no chance to customize the workflows by selecting the suitable aligners and read-counting tool along with several parameters and options. Finally, only iSmaRT [41], miARma-Seq [43], and Oasis 2 [44] allow performing differential expression analysis, miRNA target predictions, and GO/pathways enrichment analyses, while iSRAP [42] supports only a differential expression analysis module. As a final consideration, none of the tested ncRNA pipelines can analyze a comprehensive list of different classes of regulatory ncRNAs (e.g., miRNAs, piRNAs, snoRNAs, tUCRs, lncRNAs, circRNAs, and tRNA-derived ncRNAs). Indeed, they are restricted to analyzing a small set of ncRNA classes, which mainly include miRNAs, piRNAs, and snoRNAs (for further details, see Additional file 5).

## Discussion

In this paper, we have presented *RNAdetector*, a free user-friendly, stand-alone and cloud-based software for the analysis of coding and ncRNAs from RNA-Seq data of any sequenced organisms. Among its key features we cite: (1) it is freely available for non-commercial usage; (2) thanks to our Docker-based backend, *RNAdetector* can be easily installed and deployed locally in any operating system, or in public cloud environments, such as Google Cloud Platform, Microsoft Azure, and Amazon AWS, or in local clusters through Kubernetes; (3) an intuitive GUI equipped with a high-level helping guide allows researchers and users with no programming skills to rapidly analyze their RNASeq data; (4) our internal repository contains the latest updates to all supported genomes and transcriptomes; (5) it is comprehensive, and it can potentially analyze all RNA types from RNA-Seq data, including ncRNA classes that have been discovered in organisms whose genomes have been sequenced; (6) it is highly flexible since users can choose among different tools and parameters for each step of the pipeline according to user’s need; (7) our integrated reporting solution can be used to visualize and share results quickly. To show how easily users can perform an analysis of RNA-Seq data with *RNAdetector*, we chose a public small RNA-Seq project (SRP183064) from NCBI SRA, and we performed a complete analysis to identify the differentially expressed small ncRNAs and the impacted biological pathways. A short video tutorial (available as Additional file 1) shows how *RNAdetector* can be efficiently run*.* Finally, by comparing the features of *RNAdetector* against some relevant RNA-Seq and ncRNA-Seq analysis pipelines, we showed that some shortcomings are shared between the previous RNA-Seq and ncRNA-Seq pipelines. However, *RNAdetector* fills these critical gaps by combining several features with new additional ones in a single *one-stop-shop* software to simplify the user's experience allowing, at the same time, a complete analysis of RNA-Seq data.

## Conclusions

In conclusion, *RNAdetector* is a new software that fills an essential gap between the needs of biomedical and research labs to process RNA-Seq data and their common lack of technical background in performing such analysis, which usually relies on outsourcing such steps to third party bioinformatics facilities or using expensive commercial software.

### Availability and requirements

Project name: RNAdetector.

Project home page: https://rnadetector.atlas.dmi.unict.it/index.html.

Archived version: Not applicable.

Operating system(s): Windows Professional, macOS, Linux.

Programming language: JavaScript, PHP, Perl, Shell, R.

Other requirements: Docker.

License: except where otherwise noted, RNAdetector is distributed under the Creative Commons Attribution-ShareAlike 4.0 International license.

Any restrictions to use by non-academics: no restrictions.

## Supplementary Information

**Additional file 1.** Video tutorial. Short video tutorial that shows all the steps performed during the analysis of the case study small RNA-Seq datasets.

**Additional file 2.** Table with the CRC differentially expressed small ncRNAs. In this table are reported all the small ncRNAs that were found differentially expressed by RNAdetector in the CRC samples VS the adjacent normal tissue samples.

**Additional file 3.** Table with the CRC impacted biological pathways. In this table are reported all the biological pathways that were found significantly impacted in the CRC samples compared with the adjacent normal tissue samples. The analysis was performed by using MITHrIL algorithm included in RNAdetector.

**Additional file 4.** Table with feature comparisons of RNAdetector vs other RNA-Seq pipelines. The table reports the comparison of the features between RNAdetector and the 19 previously published RNA-Seq pipelines.

**Additional file 5.** Table with feature comparisons of RNAdetector vs other ncRNA-Seq pipelines. The table reports the comparison of the features between RNAdetector and the 7 previously published ncRNA-Seq pipelines.

## Data Availability

The datasets analyzed during the current study are available in the NCBI SRA repository (SRP183064) https://www.ncbi.nlm.nih.gov/sra/?term=SRP183064.

## Abbreviations

ncRNAs: Non-coding RNAs
miRNAs: MicroRNAs
piRNAs: Piwi-associated RNAs
snoRNAs: Small nuclear RNAs
tUCRs: Transcribed ultraconserved regions
lncRNAs: Long non-coding RNAs
circRNAs: Circular RNAs
tsRNAs: TRNA-derived small ncRNAs
GUI: Graphical User Interface
GTF: Gene Transfer Format
BED: Browser Extensible Data
CRC: Colon Rectal Cancer
