# Supplementary material for: RNAdetector: a free user-friendly stand-alone and cloud-based system for RNA-Seq data analysis
Source: BMC Bioinformatics. 2021 Jun 3;22:298. doi: 10.1186/s12859-021-04211-7 (PMC8173825; doi:10.1186/s12859-021-04211-7)
Supplement: Supplementary file 2 — Additional file 2. Table with the CRC differentially expressed small ncRNAs. In this table are reported all the small ncRNAs that were found differentially expressed by RNAdetector in the CRC samples VS the adjacent normal tissue samples. [file 12859_2021_4211_MOESM2_ESM.docx]

| **gene_id** | **p-value_limma** | **p-value_edger** | **FDR_limma** | **FDR_edger** | **meta_p-value** | **meta_FDR** | **log2_normalized_fold_change** |
| --- | --- | --- | --- | --- | --- | --- | --- |
| MIMAT0003215 | 1,18E-05 | 2,09E-14 | 0,000499732 | 1,79E-11 | 4,18E-14 | 3,57E-11 | 4,880170987 |
| MIMAT0026615 | 3,37E-05 | 1,15E-12 | 0,000782615 | 4,93E-10 | 2,31E-12 | 9,86E-10 | 5,300302331 |
| MIMAT0004598 | 2,69E-06 | 4,67E-12 | 0,0003729 | 1,33E-09 | 9,35E-12 | 2,66E-09 | 3,458329062 |
| MIMAT0000770 | 0,029139134 | 9,26E-12 | 0,069398216 | 1,98E-09 | 1,85E-11 | 3,96E-09 | -7,194756854 |
| MIMAT0000437 | 8,66E-06 | 1,26E-11 | 0,000499732 | 2,15E-09 | 2,51E-11 | 4,30E-09 | -4,835322132 |
| MIMAT0001536 | 5,86E-06 | 1,91E-11 | 0,000465042 | 2,72E-09 | 3,82E-11 | 5,45E-09 | 4,62771123 |
| MIMAT0000427 | 0,000154965 | 4,06E-11 | 0,001920214 | 4,44E-09 | 8,12E-11 | 8,89E-09 | -5,692386084 |
| MIMAT0000250 | 5,14E-07 | 4,16E-11 | 0,000267014 | 4,44E-09 | 8,31E-11 | 8,89E-09 | -3,118014746 |
| MIMAT0000435 | 5,57E-05 | 5,64E-11 | 0,000895157 | 5,36E-09 | 1,13E-10 | 1,07E-08 | -4,62398685 |
| MIMAT0000416_1 | 0,000413867 | 7,30E-11 | 0,003370056 | 6,24E-09 | 1,46E-10 | 1,25E-08 | -5,235100391 |
| MIMAT0000416 | 9,94E-05 | 2,73E-10 | 0,001327482 | 2,13E-08 | 5,47E-10 | 4,25E-08 | -5,309290158 |
| MIMAT0000072 | 1,07E-05 | 3,56E-10 | 0,000499732 | 2,39E-08 | 7,13E-10 | 4,77E-08 | 2,870442925 |
| MIMAT0001620 | 1,45E-05 | 3,83E-10 | 0,000537747 | 2,39E-08 | 7,65E-10 | 4,77E-08 | 3,97402926 |
| MIMAT0004764 | 0,001009148 | 3,91E-10 | 0,005869532 | 2,39E-08 | 7,81E-10 | 4,77E-08 | -6,609604962 |
| MIMAT0004601 | 1,22E-05 | 4,85E-10 | 0,000499732 | 2,76E-08 | 9,70E-10 | 5,53E-08 | -3,607127298 |
| MIMAT0000682 | 1,69E-05 | 6,04E-10 | 0,000547159 | 3,23E-08 | 1,21E-09 | 6,46E-08 | 4,280589831 |
| MIMAT0002806 | 2,65E-05 | 7,07E-10 | 0,000666333 | 3,56E-08 | 1,41E-09 | 7,11E-08 | -7,011742202 |
| MIMAT0000758 | 6,25E-07 | 8,90E-10 | 0,000267014 | 4,00E-08 | 1,78E-09 | 8,00E-08 | 4,371877391 |
| MIMAT0004552 | 8,48E-06 | 9,33E-10 | 0,000499732 | 4,00E-08 | 1,87E-09 | 8,00E-08 | -3,592017258 |
| MIMAT0004614 | 9,68E-06 | 9,39E-10 | 0,000499732 | 4,00E-08 | 1,88E-09 | 8,00E-08 | -2,740969939 |
| MIMAT0000252_2 | 0,00084069 | 9,82E-10 | 0,005208624 | 4,00E-08 | 1,96E-09 | 8,00E-08 | 4,076859939 |
| MIMAT0004958 | 0,000632997 | 1,20E-09 | 0,004295336 | 4,68E-08 | 2,41E-09 | 9,36E-08 | 4,07570966 |
| MIMAT0000318 | 2,27E-05 | 1,43E-09 | 0,000628924 | 5,30E-08 | 2,85E-09 | 1,06E-07 | 3,887672204 |
| MIMAT0000103 | 5,92E-05 | 1,73E-09 | 0,000903833 | 6,15E-08 | 3,45E-09 | 1,23E-07 | -3,388236653 |
| MIMAT0000427_1 | 0,000689392 | 2,07E-09 | 0,004499466 | 7,09E-08 | 4,15E-09 | 1,42E-07 | -5,425182057 |
| MIMAT0004549 | 2,99E-06 | 2,75E-09 | 0,0003729 | 9,03E-08 | 5,49E-09 | 1,81E-07 | 2,913633385 |
| MIMAT0003242 | 0,005865514 | 3,07E-09 | 0,022092573 | 9,73E-08 | 6,14E-09 | 1,95E-07 | 4,101143416 |
| MIMAT0000707 | 2,43E-05 | 3,72E-09 | 0,000629767 | 1,14E-07 | 7,44E-09 | 2,27E-07 | -3,781719876 |
| MIMAT0000432 | 5,98E-06 | 5,08E-09 | 0,000465042 | 1,50E-07 | 1,02E-08 | 3,00E-07 | 3,429979447 |
| MIMAT0000243 | 1,66E-05 | 8,48E-09 | 0,000547159 | 2,34E-07 | 1,70E-08 | 4,69E-07 | 3,161058641 |
| MIMAT0000259 | 4,15E-06 | 8,49E-09 | 0,000394251 | 2,34E-07 | 1,70E-08 | 4,69E-07 | 3,619512847 |
| MIMAT0001413 | 5,02E-05 | 9,39E-09 | 0,000895157 | 2,46E-07 | 1,88E-08 | 4,91E-07 | -3,425606741 |
| MIMAT0004599 | 0,000164184 | 9,48E-09 | 0,002005392 | 2,46E-07 | 1,90E-08 | 4,91E-07 | -3,732768282 |
| MIMAT0031177 | 0,00212121 | 1,21E-08 | 0,009860733 | 3,03E-07 | 2,42E-08 | 6,05E-07 | 3,734188509 |
| U72 | 2,25E-05 | 1,24E-08 | 0,000628924 | 3,03E-07 | 2,48E-08 | 6,05E-07 | 2,953751323 |
| MIMAT0000073 | 4,27E-05 | 1,51E-08 | 0,000830223 | 3,58E-07 | 3,02E-08 | 7,16E-07 | 2,955734092 |
| MIMAT0005899 | 0,001335731 | 2,24E-08 | 0,007274203 | 5,17E-07 | 4,47E-08 | 1,03E-06 | 3,219215598 |
| MIMAT0000095 | 3,57E-06 | 2,60E-08 | 0,000381524 | 5,86E-07 | 5,21E-08 | 1,17E-06 | 3,183166164 |
| MIMAT0003321 | 1,23E-05 | 3,53E-08 | 0,000499732 | 7,74E-07 | 7,06E-08 | 1,55E-06 | 2,67516031 |
| MIMAT0000261 | 1,18E-05 | 3,70E-08 | 0,000499732 | 7,90E-07 | 7,39E-08 | 1,58E-06 | 4,121599658 |
| MIMAT0004571 | 5,76E-05 | 4,55E-08 | 0,000895157 | 9,49E-07 | 9,10E-08 | 1,90E-06 | 3,275041707 |
| MIMAT0004698 | 7,89E-05 | 6,65E-08 | 0,001129784 | 1,34E-06 | 1,33E-07 | 2,69E-06 | 3,888628389 |
| MIMAT0004494 | 1,63E-06 | 6,76E-08 | 0,0003729 | 1,34E-06 | 1,35E-07 | 2,69E-06 | 2,174769461 |
| MIMAT0000443 | 3,05E-06 | 7,54E-08 | 0,0003729 | 1,47E-06 | 1,51E-07 | 2,93E-06 | -2,455595591 |
| MIMAT0000267 | 1,73E-05 | 9,58E-08 | 0,000547159 | 1,78E-06 | 1,92E-07 | 3,56E-06 | 2,479324126 |
| MIMAT0004560 | 9,35E-05 | 9,58E-08 | 0,001269107 | 1,78E-06 | 1,92E-07 | 3,56E-06 | 3,75826539 |
| MIMAT0000281 | 0,000171423 | 1,19E-07 | 0,002007765 | 2,17E-06 | 2,39E-07 | 4,35E-06 | 3,132996332 |
| ACA43 | 0,000110932 | 1,22E-07 | 0,001459184 | 2,18E-06 | 2,45E-07 | 4,36E-06 | 2,606821935 |
| HBII-95 | 2,34E-05 | 1,88E-07 | 0,000628924 | 3,28E-06 | 3,76E-07 | 6,56E-06 | 2,374065718 |
| MIMAT0004608 | 0,003809282 | 2,12E-07 | 0,015583428 | 3,58E-06 | 4,25E-07 | 7,17E-06 | 3,913016698 |
| MIMAT0000071 | 2,12E-05 | 2,14E-07 | 0,000628924 | 3,58E-06 | 4,28E-07 | 7,17E-06 | 2,202091642 |
| MIMAT0000088 | 4,19E-05 | 2,22E-07 | 0,000830223 | 3,65E-06 | 4,44E-07 | 7,31E-06 | -2,856701713 |
| U23 | 2,94E-05 | 3,28E-07 | 0,000717943 | 5,15E-06 | 6,57E-07 | 1,03E-05 | 2,489178962 |
| MIMAT0005796 | 1,04E-05 | 3,31E-07 | 0,000499732 | 5,15E-06 | 6,62E-07 | 1,03E-05 | -1,997011196 |
| MIMAT0000738 | 0,038472914 | 3,39E-07 | 0,083619707 | 5,18E-06 | 6,79E-07 | 1,04E-05 | -5,394768902 |
| HBII-99B | 3,87E-05 | 3,60E-07 | 0,000808036 | 5,39E-06 | 7,19E-07 | 1,08E-05 | 2,227930605 |
| MIMAT0000691 | 3,87E-05 | 4,65E-07 | 0,000808036 | 6,85E-06 | 9,30E-07 | 1,37E-05 | 2,366970571 |
| MIMAT0019814 | 0,000854728 | 4,96E-07 | 0,005250278 | 7,19E-06 | 9,92E-07 | 1,44E-05 | 4,407139762 |
| MIMAT0004548 | 0,005285425 | 8,61E-07 | 0,020634878 | 1,23E-05 | 1,72E-06 | 2,45E-05 | -3,624490865 |
| MIMAT0004615 | 1,40E-05 | 1,29E-06 | 0,000537747 | 1,81E-05 | 2,58E-06 | 3,62E-05 | -2,139789283 |
| MIMAT0002891 | 5,35E-05 | 1,36E-06 | 0,000895157 | 1,87E-05 | 2,72E-06 | 3,75E-05 | 2,116409492 |
| MIMAT0000457 | 0,000792642 | 1,52E-06 | 0,00494678 | 2,04E-05 | 3,05E-06 | 4,09E-05 | 2,584962501 |
| ACA58 | 5,66E-05 | 1,53E-06 | 0,000895157 | 2,04E-05 | 3,06E-06 | 4,09E-05 | 2,349584438 |
| MIMAT0003393 | 6,23E-05 | 1,57E-06 | 0,000933895 | 2,07E-05 | 3,15E-06 | 4,14E-05 | 2,066882865 |
| MIMAT0000423_1 | 3,78E-05 | 1,71E-06 | 0,000808036 | 2,22E-05 | 3,42E-06 | 4,44E-05 | -2,72131056 |
| U71d | 0,000346313 | 1,80E-06 | 0,003210561 | 2,29E-05 | 3,60E-06 | 4,59E-05 | 2,304467125 |
| MIMAT0000422_1 | 0,001260565 | 1,88E-06 | 0,006994305 | 2,37E-05 | 3,76E-06 | 4,73E-05 | -3,91753784 |
| MIMAT0004752 | 0,002766885 | 2,00E-06 | 0,012450981 | 2,45E-05 | 4,00E-06 | 4,90E-05 | -4,392317423 |
| MIMAT0004592 | 7,93E-05 | 2,01E-06 | 0,001129784 | 2,45E-05 | 4,01E-06 | 4,90E-05 | -2,644848908 |
| MIMAT0004517 | 0,000584497 | 2,10E-06 | 0,004164538 | 2,53E-05 | 4,21E-06 | 5,07E-05 | -2,961180751 |
| MIMAT0000617 | 6,57E-05 | 2,26E-06 | 0,000968933 | 2,68E-05 | 4,52E-06 | 5,36E-05 | 2,903522357 |
| MIMAT0004925 | 0,0031181 | 2,54E-06 | 0,013329878 | 2,98E-05 | 5,08E-06 | 5,95E-05 | -4,790076931 |
| MIMAT0022709 | 0,001813615 | 2,75E-06 | 0,009012776 | 3,17E-05 | 5,49E-06 | 6,34E-05 | 2,450032921 |
| MIMAT0004657 | 5,20E-05 | 2,95E-06 | 0,000895157 | 3,36E-05 | 5,89E-06 | 6,72E-05 | 3,151309323 |
| ACA31 | 0,000498657 | 3,04E-06 | 0,003841007 | 3,42E-05 | 6,07E-06 | 6,83E-05 | 2,812848584 |
| MIMAT0000085 | 0,000750337 | 3,20E-06 | 0,004752135 | 3,56E-05 | 6,41E-06 | 7,12E-05 | -2,238707235 |
| MIMAT0002874 | 0,000789096 | 4,31E-06 | 0,00494678 | 4,73E-05 | 8,63E-06 | 9,46E-05 | 2,607667031 |
| MIMAT0000460_1 | 0,015229238 | 4,48E-06 | 0,043841746 | 4,79E-05 | 8,95E-06 | 9,58E-05 | 4,010533868 |
| MIMAT0000423 | 5,46E-05 | 4,48E-06 | 0,000895157 | 4,79E-05 | 8,96E-06 | 9,58E-05 | -2,902988347 |
| MIMAT0000075 | 1,03E-05 | 7,70E-06 | 0,000499732 | 7,57E-05 | 1,03E-05 | 0,000109173 | 1,788093537 |
| MIMAT0004680 | 3,76E-05 | 5,65E-06 | 0,000808036 | 5,96E-05 | 1,13E-05 | 0,000117753 | 1,897011687 |
| MIMAT0000066 | 5,03E-05 | 5,85E-06 | 0,000895157 | 6,10E-05 | 1,17E-05 | 0,000120548 | -2,079883022 |
| MIMAT0000074_1 | 0,000175524 | 6,15E-06 | 0,002028013 | 6,33E-05 | 1,23E-05 | 0,000125177 | 1,974206381 |
| MIMAT0003260 | 0,020348597 | 6,37E-06 | 0,054031212 | 6,49E-05 | 1,27E-05 | 0,000128219 | 3,652076697 |
| MIMAT0026478 | 0,051175595 | 6,86E-06 | 0,105180609 | 6,90E-05 | 1,37E-05 | 0,000136432 | -4,349408831 |
| MIMAT0000449 | 0,000538579 | 7,03E-06 | 0,004016164 | 6,99E-05 | 1,41E-05 | 0,000138241 | 3,075625573 |
| MIMAT0004673 | 1,67E-05 | 1,68E-05 | 0,000547159 | 0,000151307 | 1,68E-05 | 0,000163343 | -1,614425802 |
| ts-112 | 3,98E-05 | 9,02E-06 | 0,000809899 | 8,77E-05 | 1,80E-05 | 0,000173395 | -2,022507399 |
| MIMAT0004507 | 0,000300237 | 1,06E-05 | 0,002917075 | 0,000102125 | 2,13E-05 | 0,000201982 | 2,077286001 |
| MIMAT0003294 | 0,000559181 | 1,08E-05 | 0,004075706 | 0,000102621 | 2,16E-05 | 0,000202986 | 2,360747344 |
| MIMAT0004928 | 0,001904566 | 1,11E-05 | 0,009270652 | 0,000104088 | 2,22E-05 | 0,000205913 | 3,172099373 |
| MIMAT0000728 | 0,000412062 | 1,17E-05 | 0,003370056 | 0,000109056 | 2,35E-05 | 0,000215767 | 3,173073233 |
| MIMAT0000222 | 0,018490016 | 1,27E-05 | 0,050347019 | 0,000116784 | 2,54E-05 | 0,000231084 | 3,850451278 |
| MIMAT0004543 | 0,025118905 | 1,61E-05 | 0,062071284 | 0,000146723 | 3,23E-05 | 0,000290357 | 3,916806064 |
| U28 | 2,35E-05 | 3,40E-05 | 0,000628924 | 0,000267081 | 3,40E-05 | 0,00030018 | 1,602810502 |
| MIMAT0000752 | 5,15E-05 | 1,70E-05 | 0,000895157 | 0,000151653 | 3,41E-05 | 0,00030018 | -2,198897632 |
| MIMAT0004504 | 0,026490563 | 1,75E-05 | 0,064216902 | 0,000154128 | 3,50E-05 | 0,00030511 | 4,273583703 |
| MIMAT0000276 | 0,00136844 | 1,84E-05 | 0,007405164 | 0,00016019 | 3,67E-05 | 0,000317144 | 2,516249751 |
| U68 | 0,00097986 | 1,88E-05 | 0,00573822 | 0,000162198 | 3,76E-05 | 0,000321151 | 2,13258564 |
| MIMAT0000091 | 0,000735726 | 1,97E-05 | 0,004694369 | 0,000168249 | 3,94E-05 | 0,000333167 | 1,964852056 |
| MIMAT0022842 | 0,000540186 | 2,07E-05 | 0,004016164 | 0,000174997 | 4,13E-05 | 0,000346563 | 2,038039361 |
| MIMAT0002875 | 0,000327624 | 2,11E-05 | 0,003078223 | 0,000176807 | 4,22E-05 | 0,000350181 | -2,533059731 |
| U17b | 0,000525021 | 2,23E-05 | 0,00397796 | 0,000185416 | 4,47E-05 | 0,000367267 | 2,147295002 |
| MIMAT0001636 | 0,007756657 | 2,38E-05 | 0,025705201 | 0,000195523 | 4,76E-05 | 0,000387322 | 2,790076931 |
| MIMAT0019981 | 0,000234174 | 2,53E-05 | 0,002502729 | 0,000204839 | 5,06E-05 | 0,00040585 | 2,921997488 |
| MIMAT0022727 | 0,000365029 | 2,54E-05 | 0,003285257 | 0,000204839 | 5,08E-05 | 0,00040585 | 1,943956417 |
| MIMAT0004588 | 0,000167369 | 2,61E-05 | 0,002007765 | 0,000208558 | 5,22E-05 | 0,000413254 | -1,722316808 |
| MIMAT0004515 | 4,61E-05 | 5,92E-05 | 0,000875505 | 0,000421999 | 5,92E-05 | 0,000464586 | -1,642651159 |
| MIMAT0000070 | 3,39E-05 | 6,08E-05 | 0,000782615 | 0,000429585 | 6,08E-05 | 0,000472543 | 1,58033653 |
| MIMAT0003888 | 0,002866664 | 3,09E-05 | 0,012587162 | 0,00024477 | 6,18E-05 | 0,000476308 | -1,955215043 |
| MIMAT0000418 | 0,000233393 | 3,47E-05 | 0,002502729 | 0,000269983 | 6,95E-05 | 0,000530323 | -1,946789094 |
| MIMAT0000275_1 | 0,000409558 | 3,53E-05 | 0,003370056 | 0,000272273 | 7,07E-05 | 0,000534908 | -2,61169385 |
| MIMAT0000087 | 0,000640119 | 3,70E-05 | 0,004309461 | 0,000282085 | 7,39E-05 | 0,000554273 | -2,568156239 |
| MIMAT0000419 | 5,55E-05 | 7,50E-05 | 0,000895157 | 0,000517438 | 7,50E-05 | 0,000557933 | -1,62956557 |
| MIMAT0000094 | 0,002739103 | 4,38E-05 | 0,012391182 | 0,000331579 | 8,76E-05 | 0,000646007 | 1,942460476 |
| U15A | 0,000185671 | 4,63E-05 | 0,002116651 | 0,000347208 | 9,26E-05 | 0,000676611 | 1,841818332 |
| U17a | 0,000227602 | 5,11E-05 | 0,002494871 | 0,000377315 | 0,000102261 | 0,00073592 | 2,067057467 |
| MIMAT0000074 | 0,000625938 | 5,16E-05 | 0,004295336 | 0,000377315 | 0,000103103 | 0,00073592 | 2,084064265 |
| MIMAT0003220 | 0,004071645 | 5,19E-05 | 0,016443564 | 0,000377315 | 0,000103727 | 0,00073592 | 2,2410081 |
| MIMAT0004502 | 0,0012808 | 5,21E-05 | 0,007019769 | 0,000377315 | 0,000104148 | 0,00073592 | -1,781420382 |
| MIMAT0022721 | 0,072275257 | 5,52E-05 | 0,137322988 | 0,000396887 | 0,000110478 | 0,000774254 | 3,843983844 |
| MIMAT0004703 | 0,000372215 | 6,22E-05 | 0,003308566 | 0,000435684 | 0,000124336 | 0,000864284 | 2,465781566 |
| MIMAT0004683 | 0,000136731 | 9,54E-05 | 0,001744847 | 0,000608506 | 0,000136731 | 0,00094278 | 1,630327805 |
| MIMAT0000090 | 0,000141476 | 9,26E-05 | 0,001778853 | 0,000599889 | 0,000141476 | 0,000967696 | 1,693202243 |
| MIMAT0000226_1 | 0,024177221 | 7,45E-05 | 0,06073869 | 0,000517438 | 0,000149066 | 0,001011522 | 2,869777423 |
| MIMAT0004671 | 0,052615145 | 7,61E-05 | 0,107621887 | 0,000518417 | 0,000152224 | 0,001020633 | 3,249196709 |
| U36A | 0,000375358 | 7,64E-05 | 0,003308566 | 0,000518417 | 0,000152797 | 0,001020633 | 1,774707688 |
| MIMAT0004978 | 0,07679004 | 8,08E-05 | 0,14342339 | 0,000544225 | 0,000161676 | 0,001069423 | 2,902702799 |
| MIMAT0000076 | 8,15E-05 | 0,000165972 | 0,001142393 | 0,00098546 | 0,000163008 | 0,001069423 | 1,751763387 |
| MIMAT0000425 | 0,000170165 | 8,19E-05 | 0,002007765 | 0,000547244 | 0,000163853 | 0,001069423 | -1,897493391 |
| MIMAT0004485 | 0,000351157 | 8,53E-05 | 0,003210561 | 0,000565634 | 0,000170682 | 0,001105557 | -1,945086919 |
| MIMAT0000680 | 8,84E-05 | 0,000462546 | 0,001219356 | 0,002234332 | 0,000176842 | 0,001127579 | 1,314448261 |
| MIMAT0000773 | 0,000123694 | 0,000177607 | 0,001602402 | 0,001019157 | 0,000177607 | 0,001127579 | -1,881737118 |
| HBII-240 | 0,001896947 | 8,90E-05 | 0,009270652 | 0,000585474 | 0,000178039 | 0,001127579 | 2,125530882 |
| MIMAT0004550 | 0,000492272 | 9,17E-05 | 0,003826295 | 0,000598445 | 0,000183383 | 0,001152887 | -2,570193041 |
| MIMAT0031175 | 0,007876959 | 9,50E-05 | 0,026003089 | 0,000608506 | 0,000189967 | 0,001185558 | -4,181897643 |
| MIMAT0019220 | 0,001685836 | 0,000101104 | 0,008579703 | 0,000640324 | 0,000202208 | 0,001252808 | 2,566346823 |
| MIMAT0001635 | 0,001463157 | 0,000109354 | 0,007722216 | 0,000687486 | 0,000218709 | 0,001345296 | 2,25947088 |
| MIMAT0003249 | 0,02973824 | 0,000113566 | 0,070343231 | 0,000708749 | 0,000227131 | 0,001387123 | 2,480525091 |
| MIMAT0000428_1 | 0,003042752 | 0,000115532 | 0,013101684 | 0,000715798 | 0,000231065 | 0,001401137 | -3,037089319 |
| MIMAT0000731 | 0,006964968 | 0,000116559 | 0,023683707 | 0,000716627 | 0,000233118 | 0,001403186 | -1,996729323 |
| MIMAT0004793 | 0,017806581 | 0,000117342 | 0,049270638 | 0,000716627 | 0,000234685 | 0,001403186 | 2,554588852 |
| MIMAT0000772 | 0,000261533 | 0,000210872 | 0,002760622 | 0,001149991 | 0,000261533 | 0,00155285 | 1,441356673 |
| MIMAT0003389 | 0,024074793 | 0,000154225 | 0,060719611 | 0,000935194 | 0,00030845 | 0,001818042 | 2,494618566 |
| MIMAT0004493 | 0,000206283 | 0,000310449 | 0,002320685 | 0,00162843 | 0,000310449 | 0,001818042 | 1,435386145 |
| MIMAT0000089 | 0,039303209 | 0,000157056 | 0,085074036 | 0,000945657 | 0,000314113 | 0,001826984 | 2,625214668 |
| U36B | 0,000318282 | 0,000181497 | 0,003057657 | 0,001034531 | 0,000318282 | 0,001832429 | 1,508109582 |
| U71b | 0,00070841 | 0,000159668 | 0,004588564 | 0,000954657 | 0,000319336 | 0,001832429 | 1,752213368 |
| MIMAT0026720 | 0,000629733 | 0,00016911 | 0,004295336 | 0,000993273 | 0,000338221 | 0,001920766 | -2,44625623 |
| MIMAT0001341 | 0,001552773 | 0,000169611 | 0,007997717 | 0,000993273 | 0,000339223 | 0,001920766 | 1,724000699 |
| MIMAT0030020 | 0,000274249 | 0,000341488 | 0,002825091 | 0,001758868 | 0,000341488 | 0,001920869 | 2,529253068 |
| MIMAT0004983 | 0,001988994 | 0,000173426 | 0,009500501 | 0,0010087 | 0,000346851 | 0,001938287 | 1,972414168 |
| MIMAT0003385 | 0,013948274 | 0,000176965 | 0,041265655 | 0,001019157 | 0,00035393 | 0,001965003 | -3,222392421 |
| MIMAT0015053 | 0,016630293 | 0,000185203 | 0,046619346 | 0,001048666 | 0,000370406 | 0,002043207 | 2,61667136 |
| ACA28 | 0,000386772 | 0,00018933 | 0,003355938 | 0,00105687 | 0,00037866 | 0,002060227 | 1,994304803 |
| MIMAT0000434 | 0,001187864 | 0,000189416 | 0,006770826 | 0,00105687 | 0,000378831 | 0,002060227 | 2,056471564 |
| MIMAT0001625 | 0,019252011 | 0,00019036 | 0,051762482 | 0,00105687 | 0,00038072 | 0,002060227 | 3,129283017 |
| MIMAT0000260 | 0,00238117 | 0,000203221 | 0,0109457 | 0,001120995 | 0,000406443 | 0,002185588 | 2,276124405 |
| ACA3-2 | 0,004027307 | 0,000211168 | 0,016396895 | 0,001149991 | 0,000422336 | 0,002256857 | 1,83051911 |
| MIMAT0000461 | 0,000400532 | 0,000437991 | 0,003357402 | 0,002159331 | 0,000437991 | 0,002325977 | -1,699073713 |
| MIMAT0004511 | 0,000221754 | 0,003678886 | 0,002462332 | 0,01220315 | 0,000443508 | 0,002340735 | -2,620237975 |
| MIMAT0000689 | 0,00032263 | 0,000449628 | 0,003064983 | 0,002196752 | 0,000449628 | 0,002358476 | -1,634989303 |
| MIMAT0000077 | 0,000291517 | 0,000461493 | 0,002914338 | 0,002234332 | 0,000461493 | 0,002405953 | -1,449365269 |
| MIMAT0003297 | 0,000295973 | 0,00049638 | 0,002914338 | 0,002384295 | 0,00049638 | 0,002563554 | -1,587692293 |
| MIMAT0002173 | 0,639576123 | 0,00024886 | 0,725248787 | 0,001346677 | 0,000497719 | 0,002563554 | 4,834692334 |
| MIMAT0000080 | 0,000269384 | 0,000975811 | 0,002808817 | 0,004195704 | 0,000538767 | 0,00274194 | -1,314627839 |
| MIMAT0000097 | 0,000280205 | 0,000628289 | 0,002852084 | 0,002919493 | 0,00056041 | 0,002835208 | -2,627655225 |
| MIMAT0018184_1 | 0,031198121 | 0,000281917 | 0,07194654 | 0,001506491 | 0,000563833 | 0,002835748 | -4,142957954 |
| MIMAT0022717 | 0,036005828 | 0,00029411 | 0,079159625 | 0,001561888 | 0,00058822 | 0,002941099 | -4,475733431 |
| MIMAT0000082_1 | 0,000296547 | 0,001556959 | 0,002914338 | 0,005969505 | 0,000593093 | 0,002948226 | -1,203183813 |
| ACA44 | 0,001496017 | 0,0003074 | 0,007799358 | 0,001622391 | 0,000614801 | 0,003038467 | 1,677167678 |
| MIMAT0000429 | 0,06653411 | 0,000321111 | 0,130474001 | 0,001674087 | 0,000642223 | 0,003155749 | -3,663333564 |
| MIMAT0000098 | 0,000653619 | 0,000672766 | 0,00434589 | 0,00310927 | 0,000672766 | 0,003284799 | -2,036394902 |
| MIMAT0004503 | 0,00072267 | 0,000338085 | 0,004645736 | 0,001751893 | 0,000676169 | 0,003284799 | 1,512450001 |
| MIMAT0000438 | 0,00045956 | 0,000697506 | 0,003638187 | 0,003206279 | 0,000697506 | 0,00336931 | -1,49562312 |
| MIMAT0004603 | 0,000352974 | 0,001513408 | 0,003210561 | 0,005881655 | 0,000705948 | 0,00339093 | -2,369827797 |
| MIMAT0004498 | 0,001823638 | 0,000358581 | 0,009012776 | 0,001835847 | 0,000717161 | 0,003425548 | 1,816799768 |
| MIMAT0004909 | 0,015859144 | 0,000366089 | 0,0450484 | 0,001863129 | 0,000732177 | 0,003477841 | 1,948594095 |
| U33 | 0,000388582 | 0,001528021 | 0,003355938 | 0,005884944 | 0,000777165 | 0,003670318 | 1,157293444 |
| U106 | 0,000902709 | 0,000390796 | 0,005359838 | 0,001977103 | 0,000781592 | 0,003670318 | 1,459789593 |
| HBII-13 | 0,000392788 | 0,000976544 | 0,003357402 | 0,004195704 | 0,000785577 | 0,003670318 | -1,255216268 |
| MIMAT0000065 | 0,000397426 | 0,005421925 | 0,003357402 | 0,017043185 | 0,000794853 | 0,003693472 | -1,042143336 |
| U20 | 0,000885629 | 0,000416345 | 0,005295195 | 0,002093972 | 0,000832691 | 0,003841462 | 1,452409293 |
| MIMAT0000093 | 0,000419604 | 0,006149475 | 0,003384543 | 0,019106453 | 0,000839208 | 0,003841462 | 1,018581386 |
| MIMAT0000718 | 0,000472444 | 0,000844338 | 0,003705869 | 0,003779628 | 0,000844338 | 0,003841462 | -1,345467652 |
| ACA8 | 0,006845223 | 0,000422336 | 0,023683707 | 0,002111681 | 0,000844672 | 0,003841462 | 1,857120044 |
| MIMAT0000424_1 | 0,000862572 | 0,000813932 | 0,005250278 | 0,003682073 | 0,000862572 | 0,003902113 | -2,399930607 |
| U64 | 0,002870756 | 0,000437738 | 0,012587162 | 0,002159331 | 0,000875476 | 0,003934278 | 1,749565631 |
| U47 | 0,001804996 | 0,000439443 | 0,009012776 | 0,002159331 | 0,000878885 | 0,003934278 | 1,480968739 |
| MIMAT0000064 | 0,000446505 | 0,001807759 | 0,003567867 | 0,006720147 | 0,00089301 | 0,003976685 | -2,372478696 |
| ACA63 | 0,000632591 | 0,000903851 | 0,004295336 | 0,003986675 | 0,000903851 | 0,003986675 | -1,498453507 |
| MIMAT0003880 | 0,000562495 | 0,000904579 | 0,004075706 | 0,003986675 | 0,000904579 | 0,003986675 | 1,40599236 |
| MIMAT0002820 | 0,00054729 | 0,00096782 | 0,004033908 | 0,004195704 | 0,00096782 | 0,004221866 | -1,460470924 |
| MIMAT0004505 | 0,000525742 | 0,001167862 | 0,00397796 | 0,004823779 | 0,001051484 | 0,004552086 | 1,285242071 |
| MIMAT0004951 | 0,000578812 | 0,001054779 | 0,004158691 | 0,004464535 | 0,001054779 | 0,004552086 | -1,551871714 |
| MIMAT0018965 | 0,032863914 | 0,000529746 | 0,074929723 | 0,00253035 | 0,001059491 | 0,004552086 | 2,781359714 |
| MIMAT0000715 | 0,001100713 | 0,000879452 | 0,006358848 | 0,003916309 | 0,001100713 | 0,004705547 | -1,299707512 |
| U13 | 0,009255704 | 0,000573584 | 0,029309728 | 0,002724523 | 0,001147168 | 0,004879743 | 1,808537556 |
| MIMAT0000430_1 | 0,003778645 | 0,000593634 | 0,015532409 | 0,002804184 | 0,001187268 | 0,005025319 | -1,993135459 |
| MIMAT0004763 | 0,001525147 | 0,000602942 | 0,007903035 | 0,002832502 | 0,001205884 | 0,005078969 | -2,453172628 |
| U43 | 0,000611772 | 0,001223454 | 0,004295336 | 0,004911047 | 0,001223454 | 0,005127711 | -1,262935455 |
| MIMAT0004586 | 0,024224437 | 0,000622736 | 0,06073869 | 0,002909502 | 0,001245471 | 0,005175687 | 1,663152949 |
| MIMAT0023712 | 0,000623504 | 0,002747575 | 0,004295336 | 0,009627773 | 0,001247008 | 0,005175687 | -1,215587243 |
| 14q(II-3) | 0,000678337 | 0,001713395 | 0,004461367 | 0,006425232 | 0,001356673 | 0,00560365 | -1,424687669 |
| MIMAT0022482 | 0,014580808 | 0,000737014 | 0,04225963 | 0,003369771 | 0,001474029 | 0,006059108 | 2,184424571 |
| MIMAT0004565 | 0,001482033 | 0,001386981 | 0,007773853 | 0,005496298 | 0,001482033 | 0,006062862 | -2,637814383 |
| MIMAT0026482 | 0,070937627 | 0,000759262 | 0,136667554 | 0,003453027 | 0,001518524 | 0,006182563 | 2,012600037 |
| MIMAT0004761 | 0,962082259 | 0,000822339 | 0,973467847 | 0,003700524 | 0,001644678 | 0,006664452 | 4,320167637 |
| U95 | 0,000865835 | 0,006495883 | 0,005250278 | 0,019978345 | 0,001731671 | 0,00698386 | -1,004914693 |
| MIMAT0010214 | 0,000883839 | 0,001784218 | 0,005295195 | 0,006661599 | 0,001767678 | 0,007095608 | -1,5334322 |
| MIMAT0000265 | 0,00180476 | 0,001708017 | 0,009012776 | 0,006425232 | 0,00180476 | 0,007210605 | -1,942190732 |
| MIMAT0000082 | 0,000912998 | 0,004816738 | 0,005383541 | 0,015424384 | 0,001825996 | 0,00726152 | -1,142439734 |
| MIMAT0004797 | 0,004603065 | 0,000934577 | 0,018305213 | 0,004077065 | 0,001869154 | 0,007398736 | -1,601675746 |
| MIMAT0004489 | 0,006955249 | 0,001033398 | 0,023683707 | 0,004417774 | 0,002066795 | 0,008143363 | 1,855414378 |
| MIMAT0004953 | 0,01580005 | 0,001043754 | 0,0450484 | 0,004439851 | 0,002087509 | 0,008187248 | -2,741466986 |
| MIMAT0000683 | 0,001908345 | 0,002124559 | 0,009270652 | 0,007796127 | 0,002124559 | 0,00829451 | -1,222968808 |
| MIMAT0000275 | 0,002841788 | 0,001078836 | 0,012587162 | 0,004543867 | 0,002157673 | 0,008385501 | -2,186413124 |
| MIMAT0003247 | 0,006745968 | 0,001092309 | 0,023638536 | 0,004578059 | 0,002184618 | 0,008451802 | -1,674829701 |
| MIMAT0000062 | 0,001418915 | 0,00222594 | 0,007630013 | 0,008064316 | 0,00222594 | 0,008569955 | -1,28804258 |
| ACA32 | 0,008153548 | 0,001117602 | 0,026607953 | 0,00466122 | 0,002235205 | 0,008569955 | 1,827503141 |
| MIMAT0004587 | 0,001131635 | 0,00355266 | 0,006493612 | 0,011865329 | 0,002263271 | 0,008638823 | -1,226562063 |
| MIMAT0015015 | 0,079493715 | 0,001166169 | 0,147754623 | 0,004823779 | 0,002332337 | 0,008862882 | 2,895302621 |
| HBII-82 | 0,016998238 | 0,001174363 | 0,047495077 | 0,00482731 | 0,002348726 | 0,008885668 | 1,89077093 |
| MIMAT0004605 | 0,041445009 | 0,001193202 | 0,088810734 | 0,00488128 | 0,002386403 | 0,008976574 | -2,251816182 |
| U77 | 0,014180165 | 0,001206539 | 0,041520689 | 0,004895099 | 0,002413078 | 0,008976574 | 1,822654058 |
| MIMAT0000458 | 0,072647087 | 0,00120803 | 0,137723413 | 0,004895099 | 0,002416061 | 0,008976574 | 1,693688375 |
| MIMAT0018205 | 0,001212354 | 0,003465891 | 0,006850544 | 0,011666682 | 0,002424707 | 0,008976574 | -1,466085105 |
| MIMAT0019820 | 0,237514864 | 0,001216291 | 0,342453978 | 0,004905323 | 0,002432581 | 0,008976574 | 2,624490865 |
| MIMAT0000067 | 0,001217874 | 0,011382139 | 0,006850544 | 0,031907308 | 0,002435749 | 0,008976574 | -0,883173187 |
| U15B | 0,001248427 | 0,002789292 | 0,006976505 | 0,009734058 | 0,002496854 | 0,009162277 | 1,16544335 |
| MIMAT0000681 | 0,001267974 | 0,007664757 | 0,006994305 | 0,022597845 | 0,002535947 | 0,00926596 | -1,043080091 |
| MIMAT0004985 | 0,036879061 | 0,001284601 | 0,080643471 | 0,005132401 | 0,002569202 | 0,009347522 | 1,985303489 |
| MIMAT0000705 | 0,002700154 | 0,002630429 | 0,012279954 | 0,009255213 | 0,002700154 | 0,009782337 | 1,234953711 |
| MIMAT0022838 | 0,022406365 | 0,001388539 | 0,058265735 | 0,005496298 | 0,002777077 | 0,010018569 | 1,657531127 |
| U14A | 0,005965551 | 0,001410926 | 0,022370815 | 0,00555918 | 0,002821853 | 0,010137328 | 1,641344971 |
| MIMAT0005792_1 | 0,001444645 | 0,003125691 | 0,007674526 | 0,010647275 | 0,00288929 | 0,010296656 | -1,3264062 |
| MIMAT0003239 | 0,001445145 | 0,003501497 | 0,007674526 | 0,011740314 | 0,00289029 | 0,010296656 | -1,165063478 |
| MIMAT0025470 | 0,026423758 | 0,001462897 | 0,064216902 | 0,005737508 | 0,002925793 | 0,010379889 | -2,722466024 |
| MIMAT0003257 | 0,007590354 | 0,001498342 | 0,025350597 | 0,005849691 | 0,002996684 | 0,010548946 | 1,840219556 |
| U79 | 0,002998121 | 0,002896145 | 0,013057099 | 0,010025118 | 0,002998121 | 0,010548946 | 1,199548678 |
| MIMAT0005923 | 0,592383334 | 0,001527104 | 0,684442906 | 0,005884944 | 0,003054208 | 0,010702244 | 4,554588852 |
| MIMAT0025477 | 0,137355022 | 0,001567646 | 0,216277245 | 0,005983648 | 0,003135292 | 0,010941528 | 2,688055994 |
| U101 | 0,001575722 | 0,00769139 | 0,008067321 | 0,022598413 | 0,003151445 | 0,010953192 | 0,938847362 |
| MIMAT0003250 | 0,096425267 | 0,001668677 | 0,166326585 | 0,006340974 | 0,003337355 | 0,011552381 | -2,492396382 |
| ACA50 | 0,014132789 | 0,001702448 | 0,041520689 | 0,006425232 | 0,003404895 | 0,011738651 | 1,761612601 |
| U49B | 0,003515242 | 0,002412609 | 0,014705614 | 0,008667144 | 0,003515242 | 0,01207041 | 1,360452362 |
| MIMAT0018972 | 0,001765566 | 0,035277837 | 0,0089323 | 0,075325338 | 0,003531131 | 0,012076469 | -2,554588852 |
| HBII-180C | 0,014241247 | 0,001890018 | 0,041557224 | 0,006995522 | 0,003780037 | 0,012876221 | 1,549216839 |
| MIMAT0022471 | 0,001962763 | 0,005235744 | 0,009481145 | 0,016579855 | 0,003925527 | 0,013318751 | -2,076350886 |
| MIMAT0000067_1 | 0,001974307 | 0,010520468 | 0,009483326 | 0,029784767 | 0,003948613 | 0,013344127 | -1,018910059 |
| MIMAT0000684 | 0,00327565 | 0,004041741 | 0,013864757 | 0,013291109 | 0,004041741 | 0,013605073 | -1,236137513 |
| MIMAT0000083 | 0,002057122 | 0,019756242 | 0,009771327 | 0,047987463 | 0,004114243 | 0,013776035 | -0,873262426 |
| SNORD121A | 0,010997788 | 0,002062377 | 0,033582532 | 0,007600571 | 0,004124754 | 0,013776035 | 1,514573173 |
| MIMAT0004597 | 0,002105908 | 0,008093557 | 0,009860733 | 0,023617717 | 0,004211817 | 0,014012075 | -1,107190784 |
| MIMAT0000244 | 0,002122076 | 0,008692062 | 0,009860733 | 0,025277934 | 0,004244152 | 0,014064921 | -1,04510803 |
| MIMAT0022258 | 0,018765844 | 0,00213511 | 0,050794473 | 0,007801363 | 0,00427022 | 0,014096671 | 2,584962501 |
| MIMAT0003258 | 0,005817796 | 0,002223525 | 0,022009804 | 0,008064316 | 0,00444705 | 0,014612903 | 1,525256255 |
| U24 | 0,002813222 | 0,004460781 | 0,012587162 | 0,014501778 | 0,004460781 | 0,014612903 | 1,097241646 |
| MIMAT0000510 | 0,002251799 | 0,006738463 | 0,010406964 | 0,020430445 | 0,004503599 | 0,014696858 | -1,087862116 |
| MIMAT0000272 | 0,053139314 | 0,002410326 | 0,10817646 | 0,008667144 | 0,004820653 | 0,015671703 | 3,077244765 |
| U57 | 0,004954716 | 0,004269696 | 0,019612416 | 0,013986934 | 0,004954716 | 0,016046522 | 1,139953253 |
| MIMAT0001618 | 0,003706346 | 0,004995975 | 0,015308822 | 0,015938651 | 0,004995975 | 0,016119088 | 1,256013978 |
| MIMAT0003338 | 0,002521416 | 0,014261039 | 0,0115284 | 0,037291176 | 0,005042832 | 0,016209104 | 1,004063159 |
| 14q(II-1) | 0,003461165 | 0,005074022 | 0,014577812 | 0,016127469 | 0,005074022 | 0,016248274 | -1,351763324 |
| MIMAT0000721 | 0,048659505 | 0,002572938 | 0,100980284 | 0,009204444 | 0,005145876 | 0,016416881 | 1,591095114 |
| MIMAT0002819 | 0,006980461 | 0,002591739 | 0,023683707 | 0,00923307 | 0,005183478 | 0,016475367 | -1,342195536 |
| MIMAT0004801 | 0,006309034 | 0,002606531 | 0,022433388 | 0,009247237 | 0,005213062 | 0,01650803 | 1,215108695 |
| MIMAT0010251 | 0,113937775 | 0,002627285 | 0,190268452 | 0,009255213 | 0,005254569 | 0,016578069 | 3,211504105 |
| MIMAT0002872 | 0,010177743 | 0,002824992 | 0,031465618 | 0,009818569 | 0,005649984 | 0,01773435 | 1,499493159 |
| U38B | 0,002831273 | 0,007992623 | 0,012587162 | 0,023403056 | 0,005662547 | 0,01773435 | 0,972979076 |
| MIMAT0022726 | 0,005702972 | 0,003696627 | 0,021703985 | 0,01220315 | 0,005702972 | 0,01779577 | -1,362983163 |
| MIMAT0000226 | 0,106700699 | 0,002946478 | 0,181092572 | 0,01015822 | 0,005892956 | 0,018321735 | 2,437063806 |
| snR38C | 0,015221625 | 0,002978736 | 0,043841746 | 0,010226214 | 0,005957472 | 0,018455213 | 1,426442263 |
| MIMAT0014996 | 0,489920595 | 0,002990121 | 0,595849373 | 0,010226214 | 0,005980242 | 0,01845887 | 4,232660757 |
| MIMAT0000440 | 0,003008478 | 0,019695597 | 0,013057099 | 0,047976453 | 0,006016955 | 0,018505384 | 0,900033028 |
| MIMAT0000063 | 0,003049398 | 0,025534201 | 0,013101684 | 0,05790913 | 0,006098796 | 0,018689858 | -0,847030893 |
| MIMAT0000460 | 0,07511335 | 0,003195428 | 0,141457961 | 0,010841632 | 0,006390857 | 0,019514938 | 3,014873276 |
| MIMAT0002813 | 0,027355377 | 0,003268812 | 0,065514978 | 0,011046776 | 0,006537624 | 0,01982527 | 1,484024977 |
| MIMAT0000703 | 0,00326943 | 0,039013115 | 0,013864757 | 0,08115867 | 0,006538861 | 0,01982527 | -0,754391134 |
| MIMAT0026472 | 0,003525907 | 0,023852662 | 0,014705614 | 0,05482265 | 0,007051815 | 0,021304952 | -1,971985624 |
| MIMAT0026479 | 0,003609424 | 0,008772786 | 0,01498086 | 0,025426209 | 0,007218847 | 0,021732797 | -1,343954401 |
| HBII-234 | 0,009773209 | 0,003688842 | 0,030565283 | 0,01220315 | 0,007377683 | 0,022133049 | 1,279115011 |
| MIMAT0002873 | 0,007484448 | 0,005710235 | 0,025094915 | 0,017818433 | 0,007484448 | 0,022374837 | 1,333423734 |
| MIMAT0000717 | 0,007434336 | 0,007664766 | 0,025025027 | 0,022597845 | 0,007664766 | 0,022834059 | -1,254929356 |
| MIMAT0000262 | 0,004077235 | 0,377933152 | 0,016443564 | 0,483008736 | 0,008154469 | 0,02420858 | -1,078379293 |
| U16 | 0,008248012 | 0,004650291 | 0,02681388 | 0,015003768 | 0,008248012 | 0,024401559 | 1,221364128 |
| MIMAT0004602 | 0,004177803 | 0,010681954 | 0,016770055 | 0,030142148 | 0,008355606 | 0,024634633 | -1,208430747 |
| MIMAT0004767 | 0,02367362 | 0,004308925 | 0,060239175 | 0,014061568 | 0,00861785 | 0,025320487 | -1,639597757 |
| MIMAT0005882 | 0,008655016 | 0,006660428 | 0,027819693 | 0,020265716 | 0,008655016 | 0,025342597 | 1,33219643 |
| HBII-316 | 0,008841817 | 0,006167697 | 0,028208037 | 0,019106453 | 0,008841817 | 0,025744506 | 1,215977582 |
| MIMAT0004809 | 0,004426248 | 0,011431631 | 0,01768431 | 0,031941323 | 0,008852497 | 0,025744506 | -1,150882554 |
| ACA33 | 0,019386378 | 0,004627124 | 0,051960355 | 0,014985573 | 0,009254248 | 0,026821635 | 1,38466385 |
| ACA26 | 0,012489823 | 0,004753839 | 0,037338458 | 0,015280196 | 0,009507677 | 0,027463054 | 1,485426827 |
| U83A | 0,00979519 | 0,009348565 | 0,030565283 | 0,026643411 | 0,00979519 | 0,028198275 | 1,088900505 |
| MIMAT0000104 | 0,005258124 | 0,014417278 | 0,020630018 | 0,037581623 | 0,010516248 | 0,030082567 | 0,961831736 |
| MIMAT0001343 | 0,005260051 | 0,016849247 | 0,020630018 | 0,042531576 | 0,010520103 | 0,030082567 | 0,862710778 |
| MIMAT0026621 | 0,005341287 | 0,016863397 | 0,020758185 | 0,042531576 | 0,010682575 | 0,030445338 | -1,383704292 |
| U51 | 0,025721203 | 0,005403664 | 0,063013263 | 0,017043185 | 0,010807329 | 0,030698558 | 1,261213793 |
| MIMAT0004491 | 0,008073635 | 0,010965262 | 0,026549839 | 0,030839799 | 0,010965262 | 0,031044036 | 1,235216462 |
| MIMAT0002888 | 0,005655075 | 0,021166173 | 0,021703985 | 0,049991927 | 0,011310151 | 0,031758957 | 0,94403957 |
| U44 | 0,005658071 | 0,01264736 | 0,021703985 | 0,034328549 | 0,011316141 | 0,031758957 | -0,980612835 |
| MIMAT0005930 | 0,190077614 | 0,005664609 | 0,28411951 | 0,017740809 | 0,011329219 | 0,031758957 | 2,157541277 |
| MIMAT0000253 | 0,005708627 | 0,024903236 | 0,021703985 | 0,056864665 | 0,011417255 | 0,031813659 | -0,971376526 |
| MIMAT0016895 | 0,005711575 | 0,027305594 | 0,021703985 | 0,061613102 | 0,01142315 | 0,031813659 | -0,872377497 |
| 14q(II-12) | 0,006886575 | 0,011682495 | 0,023683707 | 0,032399799 | 0,011682495 | 0,032430304 | -1,270357747 |
| MIMAT0000092 | 0,009938118 | 0,011925965 | 0,030898513 | 0,03289258 | 0,011925965 | 0,032999028 | 1,029590319 |
| MIMAT0000693 | 0,006020173 | 0,028986392 | 0,022433388 | 0,064205608 | 0,012040346 | 0,033208052 | -0,787110389 |
| tRFdb-3033a | 0,006323329 | 0,014725645 | 0,022433388 | 0,038037542 | 0,012646659 | 0,033685025 | -1,150941898 |
| tRFdb-3033a.1 | 0,006323329 | 0,014633547 | 0,022433388 | 0,03791419 | 0,012646659 | 0,033685025 | -1,150941898 |
| tRFdb-3033a.2 | 0,006323329 | 0,014618056 | 0,022433388 | 0,03791419 | 0,012646659 | 0,033685025 | -1,150941898 |
| tRFdb-3033a.3 | 0,006323329 | 0,013992166 | 0,022433388 | 0,037272568 | 0,012646659 | 0,033685025 | -1,150941898 |
| tRFdb-3033a.4 | 0,006323329 | 0,014069508 | 0,022433388 | 0,037272568 | 0,012646659 | 0,033685025 | -1,150941898 |
| tRFdb-3033a.5 | 0,006323329 | 0,014034276 | 0,022433388 | 0,037272568 | 0,012646659 | 0,033685025 | -1,150941898 |
| tRFdb-3033a.6 | 0,006323329 | 0,014220366 | 0,022433388 | 0,037291176 | 0,012646659 | 0,033685025 | -1,150941898 |
| tRFdb-3033a.7 | 0,006323329 | 0,015041871 | 0,022433388 | 0,0384958 | 0,012646659 | 0,033685025 | -1,150941898 |
| tRFdb-3033a.8 | 0,006323329 | 0,01508315 | 0,022433388 | 0,0384958 | 0,012646659 | 0,033685025 | -1,150941898 |
| tRFdb-3033a.9 | 0,006323329 | 0,015023959 | 0,022433388 | 0,0384958 | 0,012646659 | 0,033685025 | -1,150941898 |
| tRFdb-3033a.10 | 0,006323329 | 0,014883599 | 0,022433388 | 0,03832975 | 0,012646659 | 0,033685025 | -1,150941898 |
| MIMAT0004556 | 0,043684145 | 0,006440515 | 0,092955895 | 0,019879569 | 0,012881031 | 0,034147834 | -1,411589764 |
| MIMAT0000750 | 0,006450146 | 0,038967651 | 0,02278874 | 0,08115867 | 0,012900293 | 0,034147834 | -0,791291163 |
| ACA18 | 0,014166191 | 0,006555874 | 0,041520689 | 0,020066997 | 0,013111749 | 0,034576979 | 1,308027278 |
| U45C | 0,024511718 | 0,006571648 | 0,061279295 | 0,020066997 | 0,013143296 | 0,034576979 | 1,250491124 |
| MIMAT0004555 | 0,013293835 | 0,012222167 | 0,039603585 | 0,033493438 | 0,013293835 | 0,034865733 | -1,04075976 |
| HBII-210 | 0,006690642 | 0,016913491 | 0,023541148 | 0,042532456 | 0,013381284 | 0,034987761 | -0,938012269 |
| ACA20 | 0,029782748 | 0,006766221 | 0,070343231 | 0,020441638 | 0,013532442 | 0,035184395 | 1,412125904 |
| U50 | 0,006777392 | 0,017875303 | 0,023651713 | 0,044428443 | 0,013554783 | 0,035184395 | 0,869727535 |
| MIMAT0003161 | 0,030142642 | 0,006789971 | 0,070802085 | 0,020441638 | 0,013579942 | 0,035184395 | 1,3408283 |
| MIMAT0000433 | 0,006944086 | 0,018140844 | 0,023683707 | 0,04469862 | 0,013888172 | 0,035818453 | 1,220353536 |
| U31 | 0,006954226 | 0,024940643 | 0,023683707 | 0,056864665 | 0,013908452 | 0,035818453 | -0,847607713 |
| MIMAT0004774 | 0,00772207 | 0,013969932 | 0,025690155 | 0,037272568 | 0,013969932 | 0,035868745 | 1,038882661 |
| U83B | 0,008692415 | 0,014080748 | 0,027835261 | 0,037272568 | 0,014080748 | 0,035964668 | 0,932547818 |
| MIMAT0026476 | 0,131513447 | 0,007065791 | 0,210175695 | 0,021126099 | 0,014131581 | 0,035964668 | 2,885751829 |
| MIMAT0004693 | 0,015816157 | 0,007066742 | 0,0450484 | 0,021126099 | 0,014133484 | 0,035964668 | 1,260460462 |
| MIMAT0004613 | 0,017987341 | 0,00713518 | 0,049610247 | 0,021256374 | 0,014270361 | 0,036205218 | 1,553253641 |
| U42B | 0,007299153 | 0,017000117 | 0,024667099 | 0,042624927 | 0,014598307 | 0,03692767 | -0,880057176 |
| MIMAT0005584 | 0,127256252 | 0,007462123 | 0,206459384 | 0,022153177 | 0,014924245 | 0,037640796 | 1,761840263 |
| MIMAT0004945 | 0,008116171 | 0,036492577 | 0,026587458 | 0,077422217 | 0,016232343 | 0,040819567 | -0,785971052 |
| U48 | 0,00842168 | 0,018345205 | 0,02727476 | 0,045072272 | 0,01684336 | 0,042231886 | -0,91799878 |
| MIMAT0004927 | 0,017075767 | 0,011650322 | 0,04755629 | 0,032399799 | 0,017075767 | 0,042618036 | 1,218534946 |
| MIMAT0000081 | 0,00854853 | 0,050948823 | 0,027581106 | 0,100603334 | 0,01709706 | 0,042618036 | 0,721333924 |
| MIMAT0021086 | 0,125587452 | 0,008817095 | 0,204528137 | 0,025468299 | 0,017634191 | 0,043829166 | 2 |
| MIMAT0019693 | 0,06102464 | 0,008888385 | 0,121748611 | 0,025587775 | 0,01777677 | 0,044055473 | 2,032421478 |
| MIMAT0022692 | 0,015566069 | 0,017981841 | 0,044661036 | 0,04445251 | 0,017981841 | 0,044245923 | 1,674599713 |
| MIMAT0018204 | 0,011113722 | 0,017988969 | 0,033695858 | 0,04445251 | 0,017988969 | 0,044245923 | -1,934904972 |
| HBII-180A | 0,082724048 | 0,009025443 | 0,151888706 | 0,025895148 | 0,018050887 | 0,044245923 | 1,476438044 |
| MIMAT0004678 | 0,009030308 | 0,023564598 | 0,028702281 | 0,054448706 | 0,018060616 | 0,044245923 | -1,100603941 |
| MIMAT0001541 | 0,217149928 | 0,009081104 | 0,316831379 | 0,025967706 | 0,018162208 | 0,044367681 | 2,612518223 |
| HBII-108B | 0,018773162 | 0,018576613 | 0,050794473 | 0,04551004 | 0,018773162 | 0,045729497 | 1,036246566 |
| MIMAT0000762 | 0,009463201 | 0,029177383 | 0,029856224 | 0,064461661 | 0,018926402 | 0,045971799 | -0,880329769 |
| U29 | 0,009527747 | 0,046291054 | 0,029949353 | 0,094011524 | 0,019055495 | 0,046154243 | -0,756232442 |
| MIMAT0004584 | 0,010194124 | 0,064728636 | 0,031465618 | 0,121366193 | 0,020388249 | 0,049242804 | 0,695652891 |
| MIMAT0004495 | 0,017685018 | 0,020693441 | 0,049093151 | 0,049283823 | 0,020693441 | 0,049839133 | -1,031197688 |
| MIMAT0003273 | 0,020762108 | 0,020776827 | 0,054788896 | 0,049344964 | 0,020776827 | 0,049899402 | -1,86507042 |
| MIMAT0000280 | 0,09444834 | 0,010432998 | 0,163799859 | 0,029635261 | 0,020865996 | 0,049973185 | 1,526545814 |
| MIMAT0004488 | 0,021162731 | 0,01380489 | 0,055503483 | 0,037116921 | 0,021162731 | 0,050542278 | 1,352516415 |
| HBII-85-8 | 0,010690329 | 0,108795395 | 0,032873533 | 0,183105308 | 0,021380658 | 0,050920508 | -1,117925725 |
| MIMAT0004484 | 0,010727153 | 0,08157282 | 0,032873533 | 0,143507738 | 0,021454306 | 0,050953976 | -0,630289395 |
| MIMAT0018186 | 0,011090321 | 0,042458896 | 0,033695858 | 0,087265278 | 0,022180642 | 0,0525331 | -1,02727293 |
| MIMAT0000459 | 0,020873989 | 0,022422987 | 0,054914648 | 0,052525078 | 0,022422987 | 0,052960369 | -1,115100977 |
| MIMAT0004672 | 0,011473376 | 0,039530966 | 0,03466338 | 0,081837714 | 0,022946752 | 0,054048135 | 0,757333574 |
| snR38B | 0,03516146 | 0,011709401 | 0,078343153 | 0,032399799 | 0,023418802 | 0,054799098 | 1,213955584 |
| MIMAT0000254 | 0,023457859 | 0,01232358 | 0,059870059 | 0,033663455 | 0,023457859 | 0,054799098 | -1,251209191 |
| U76 | 0,018373556 | 0,023626281 | 0,050189746 | 0,054448706 | 0,023626281 | 0,055042153 | 0,920565533 |
| MIMAT0004497 | 0,018117458 | 0,024386165 | 0,049709999 | 0,055898582 | 0,024386165 | 0,056559926 | -0,87182379 |
| ACA46 | 0,079746997 | 0,012205037 | 0,147903866 | 0,033493438 | 0,024410073 | 0,056559926 | 1,459431619 |
| MIMAT0022833 | 0,0348872 | 0,012373541 | 0,078290172 | 0,033692285 | 0,024747082 | 0,057149356 | -1,807354922 |
| U82 | 0,012399071 | 0,034197967 | 0,037197212 | 0,073650533 | 0,024798142 | 0,057149356 | -0,785231823 |
| MIMAT0005794 | 0,014293492 | 0,025015213 | 0,041567808 | 0,056882998 | 0,025015213 | 0,057494643 | -0,90749588 |
| MIMAT0007885 | 0,75894856 | 0,012863873 | 0,825573815 | 0,034805733 | 0,025727747 | 0,058973789 | 3,475733431 |
| MIMAT0022472 | 0,406503426 | 0,013031353 | 0,516434515 | 0,035147656 | 0,026062706 | 0,059581855 | -3,189824559 |
| snR39B | 0,013929485 | 0,040433571 | 0,041265655 | 0,083504115 | 0,027858969 | 0,06351845 | -0,807171772 |
| MIMAT0000454 | 0,066989288 | 0,014192215 | 0,131065999 | 0,037291176 | 0,02838443 | 0,064544382 | -2,619896291 |
| MIMAT0003244 | 0,111753095 | 0,014262239 | 0,188141989 | 0,037291176 | 0,028524478 | 0,064690793 | 1,438573014 |
| ACA57 | 0,02995947 | 0,017342636 | 0,070565694 | 0,043230185 | 0,02995947 | 0,067765468 | 1,112921513 |
| MIMAT0004949 | 0,223254657 | 0,015744728 | 0,324630496 | 0,04006471 | 0,031489456 | 0,071038219 | 1,499571009 |
| MIMAT0005825 | 0,015998685 | 0,046879086 | 0,045294291 | 0,094609196 | 0,03199737 | 0,071994083 | -0,867053506 |
| MIMAT0018183 | 0,016067815 | 0,056304661 | 0,045339875 | 0,108669266 | 0,032135631 | 0,072115392 | -0,83588062 |
| MIMAT0005874 | 0,032350325 | 0,02023608 | 0,073955957 | 0,048503171 | 0,032350325 | 0,072407141 | 1,080170349 |
| MIMAT0002871 | 0,016332337 | 0,052494452 | 0,045934698 | 0,102706537 | 0,032664674 | 0,072919833 | 0,789229423 |
| MIMAT0000453 | 0,134898185 | 0,016489138 | 0,213193989 | 0,04183446 | 0,032978276 | 0,073428193 | 1,401740677 |
| U60 | 0,021896589 | 0,034143758 | 0,05725255 | 0,073650533 | 0,034143758 | 0,075825748 | 0,843930992 |
| U58C | 0,060170379 | 0,017186017 | 0,120654656 | 0,042965042 | 0,034372033 | 0,076134944 | 1,028149942 |
| U45A | 0,035090931 | 0,021773348 | 0,078343153 | 0,051284333 | 0,035090931 | 0,077526475 | 0,998029105 |
| MIMAT0009198 | 0,018139789 | 0,04718291 | 0,049709999 | 0,094698093 | 0,036279578 | 0,079945978 | 1,014451156 |
| MIMAT0000646 | 0,019638737 | 0,036380393 | 0,052472251 | 0,077376209 | 0,036380393 | 0,079962047 | 1,11451704 |
| MIMAT0027519 | 0,037474705 | 0,019909526 | 0,081736921 | 0,048086567 | 0,037474705 | 0,082156084 | -2,736965594 |
| MIMAT0004509 | 0,018860218 | 0,088674581 | 0,050869041 | 0,154099119 | 0,037720435 | 0,082483305 | 0,615199731 |
| MIMAT0000716 | 0,026779376 | 0,037960988 | 0,064496808 | 0,079746055 | 0,037960988 | 0,082797563 | -1,257157839 |
| MIMAT0004950 | 0,736990894 | 0,019068451 | 0,804745003 | 0,046581501 | 0,038136901 | 0,082969594 | 2,044394119 |
| MIMAT0017988 | 0,020460451 | 0,03896194 | 0,054160017 | 0,08115867 | 0,03896194 | 0,084549388 | 1,062735755 |
| MIMAT0001532 | 0,939152849 | 0,019816633 | 0,953652833 | 0,047997795 | 0,039633267 | 0,085788463 | 2,754887502 |
| U78 | 0,039909116 | 0,035169716 | 0,085950362 | 0,075325338 | 0,039909116 | 0,086167409 | 0,843560766 |
| U73a | 0,020022993 | 0,065925799 | 0,053332272 | 0,123011494 | 0,040045987 | 0,086245135 | -0,67361659 |
| MIMAT0026721 | 0,825682144 | 0,020203645 | 0,860924675 | 0,048503171 | 0,040407289 | 0,086795147 | 2,115477217 |
| MIMAT0018068 | 0,063738527 | 0,020252201 | 0,126441857 | 0,048503171 | 0,040504402 | 0,086795147 | 1,525461489 |
| MIMAT0000730 | 0,130277761 | 0,020500916 | 0,20898215 | 0,048961684 | 0,041001831 | 0,087641415 | 1,21342664 |
| MIMAT0003327 | 0,885353785 | 0,020998108 | 0,909828709 | 0,049732361 | 0,041996216 | 0,089543055 | 2,289506617 |
| MIMAT0016888 | 0,035877493 | 0,042114096 | 0,079159625 | 0,086765186 | 0,042114096 | 0,089571025 | 0,879201856 |
| MIMAT0002175 | 0,026929048 | 0,043964944 | 0,0646751 | 0,089928294 | 0,043964944 | 0,093275501 | -1,086100737 |
| MIMAT0026475 | 0,085124959 | 0,022265161 | 0,155849764 | 0,052298662 | 0,044530323 | 0,094241153 | 1,230005605 |
| MIMAT0000450 | 0,022420382 | 0,061849298 | 0,058265735 | 0,117513665 | 0,044840764 | 0,094663836 | -0,951825827 |
| ACA48 | 0,022513728 | 0,053235021 | 0,058331022 | 0,103917678 | 0,045027455 | 0,094823829 | 0,832890014 |
| MIMAT0000759 | 0,022739266 | 0,080773784 | 0,05873738 | 0,143093849 | 0,045478533 | 0,095282302 | 0,672725835 |
| MIMAT0001627 | 0,04552291 | 0,030586234 | 0,096341803 | 0,067400078 | 0,04552291 | 0,095282302 | -1,116343961 |
| MIMAT0027479 | 0,242278766 | 0,022789744 | 0,344570935 | 0,053238336 | 0,045579487 | 0,095282302 | 1,547487795 |
| MIMAT0011777 | 0,030880895 | 0,045774248 | 0,071747732 | 0,093183291 | 0,045774248 | 0,095321271 | 0,880039376 |
| HBII-85-16 | 0,02293203 | 0,296124751 | 0,059056885 | 0,393987307 | 0,04586406 | 0,095321271 | -0,827441687 |
| MIMAT0019952 | 0,09060254 | 0,022966294 | 0,163638689 | 0,053504582 | 0,045932589 | 0,095321271 | 1,405256478 |
| HBII-85-2 | 0,023161599 | 0,222117409 | 0,059468971 | 0,313383473 | 0,046323198 | 0,095899115 | -0,885995371 |
| HBI-43 | 0,030757521 | 0,046599328 | 0,071694386 | 0,094413331 | 0,046599328 | 0,09614175 | 0,842540102 |
| U22 | 0,023332647 | 0,052212373 | 0,059728782 | 0,102388942 | 0,046665294 | 0,09614175 | 0,760150382 |
| U53 | 0,05463311 | 0,023474781 | 0,110690305 | 0,054448706 | 0,046949561 | 0,096424241 | 1,046833165 |
| MIMAT0000092_1 | 0,046017951 | 0,047027963 | 0,096654896 | 0,094609196 | 0,047027963 | 0,096424241 | 0,861412221 |
| MIMAT0002818 | 0,059116995 | 0,023570761 | 0,118929484 | 0,054448706 | 0,047141521 | 0,096425839 | 1,505235308 |
| MIMAT0000062_2 | 0,023743394 | 0,094924134 | 0,060239175 | 0,163959868 | 0,047486788 | 0,096900248 | -0,608969156 |
| MIMAT0000764 | 0,023964949 | 0,066037749 | 0,060621395 | 0,123011494 | 0,047929899 | 0,097571579 | 0,673031229 |
| MIMAT0005901 | 0,048467706 | 0,039004375 | 0,100826979 | 0,08115867 | 0,048467706 | 0,098432039 | -0,948925815 |
| MIMAT0004776 | 0,030317184 | 0,049127653 | 0,071016966 | 0,097912722 | 0,049127653 | 0,099535885 | -0,987616276 |
| MIMAT0000227 | 0,024723463 | 0,13809321 | 0,061628458 | 0,215281764 | 0,049446926 | 0,099945915 | -0,536898508 |
| MIMAT0004674 | 0,024847682 | 0,099216007 | 0,061758047 | 0,169659373 | 0,049695365 | 0,10006205 | -0,69366876 |
| MIMAT0027103 | 0,049738446 | 0,033519605 | 0,102969422 | 0,072739245 | 0,049738446 | 0,10006205 | -1,167841687 |
| MIMAT0017982 | 0,024997892 | 0,082196148 | 0,061951298 | 0,144307406 | 0,049995784 | 0,100343652 | -0,930459067 |
