# Supplementary material for: RNAdetector: a free user-friendly stand-alone and cloud-based system for RNA-Seq data analysis
Source: BMC Bioinformatics. 2021 Jun 3;22:298. doi: 10.1186/s12859-021-04211-7 (PMC8173825; doi:10.1186/s12859-021-04211-7)
Supplement: Supplementary file 3 — Additional file 3. Table with the CRC impacted biological pathways. In this table are reported all the biological pathways that were found significantly impacted in the CRC samples compared with the adjacent normal tissue samples. The analysis was performed by using MITHrIL algorithm included in RNAdetector. [file 12859_2021_4211_MOESM3_ESM.docx]

| **# Pathway Id** | **Pathway Name** | **Raw Accumulator** | **Impact Factor** | **Corrected Accumulator** | **pValue** | **Adjusted pValue** |
| --- | --- | --- | --- | --- | --- | --- |
| path:hsa04915 | Estrogen signaling pathway | 26,7047464 | 141,5919455 | 29,37020617 | 0 | 0 |
| path:hsa04152 | AMPK signaling pathway | 22,29944583 | 111,1487884 | 23,68710986 | 0 | 0 |
| path:hsa04630 | JAK-STAT signaling pathway | 21,07154089 | 142,6238189 | 23,3672053 | 0 | 0 |
| path:hsa04010 | MAPK signaling pathway | 16,53856412 | 175,5042568 | 20,62252326 | 0 | 0 |
| path:hsa04914 | Progesterone-mediated oocyte maturation | 16,51152087 | 84,18542026 | 18,099952 | 0 | 0 |
| path:hsa04926 | Relaxin signaling pathway | 14,91872077 | 161,7895825 | 18,06528308 | 0 | 0 |
| path:hsa04066 | HIF-1 signaling pathway | 14,64752246 | 152,0335168 | 17,61437649 | 0 | 0 |
| path:hsa04151 | PI3K-Akt signaling pathway | 11,83243342 | 184,7552123 | 16,60656147 | 0 | 0 |
| path:hsa04530 | Tight junction | -17,40251698 | 121,0519833 | -16,21177977 | 0 | 0 |
| path:hsa04140 | Autophagy - animal | 13,57336799 | 114,2792721 | 15,97382395 | 0 | 0 |
| path:hsa04668 | TNF signaling pathway | 13,6290655 | 153,6970547 | 15,15532845 | 0 | 0 |
| path:hsa04550 | Signaling pathways regulating pluripotency of stem cells | 12,39242805 | 163,5696817 | 14,93880354 | 0 | 0 |
| path:hsa04620 | Toll-like receptor signaling pathway | 11,75419672 | 158,3799077 | 13,83452509 | 0 | 0 |
| path:hsa04014 | Ras signaling pathway | 9,570188923 | 134,6365584 | 13,28883801 | 0 | 0 |
| path:hsa04150 | mTOR signaling pathway | 10,93593111 | 137,2405076 | 13,08889722 | 0 | 0 |
| path:hsa04115 | p53 signaling pathway | 11,51974924 | 133,8234914 | 13,04525948 | 0 | 0 |
| path:hsa04810 | Regulation of actin cytoskeleton | -15,50441431 | 104,3613291 | -12,81017753 | 0 | 0 |
| path:hsa04210 | Apoptosis | 10,52923471 | 162,3983766 | 12,72988821 | 0 | 0 |
| path:hsa04923 | Regulation of lipolysis in adipocytes | 11,34746219 | 38,72952459 | 11,99015355 | 0 | 0 |
| path:hsa04012 | ErbB signaling pathway | 8,814007172 | 152,4283396 | 11,84885009 | 0 | 0 |
| path:hsa04662 | B cell receptor signaling pathway | 9,74988029 | 149,0742261 | 11,75392835 | 0 | 0 |
| path:hsa04919 | Thyroid hormone signaling pathway | 8,995811995 | 182,1227329 | 11,66511078 | 0 | 0 |
| path:hsa04211 | Longevity regulating pathway | 10,19575186 | 153,8382119 | 11,55160155 | 0 | 0 |
| path:hsa04380 | Osteoclast differentiation | 9,521540344 | 174,9962572 | 11,44175005 | 0 | 0 |
| path:hsa04072 | Phospholipase D signaling pathway | 8,055705495 | 81,67978489 | 10,44888945 | 0 | 0 |
| path:hsa04660 | T cell receptor signaling pathway | 7,396780965 | 154,9192642 | 10,01976255 | 0 | 0 |
| path:hsa04213 | Longevity regulating pathway - multiple species | 8,677206041 | 103,9274246 | 9,856324468 | 0 | 0 |
| path:hsa04935 | Growth hormone synthesis, secretion and action | 6,762697436 | 140,6288458 | 9,602942328 | 0 | 0 |
| path:hsa04070 | Phosphatidylinositol signaling system | 8,004588157 | 73,21778709 | 9,083096511 | 0 | 0 |
| path:hsa04062 | Chemokine signaling pathway | -12,85590922 | 168,7593354 | -9,035746942 | 0 | 0 |
| path:hsa04725 | Cholinergic synapse | 7,288512991 | 79,17120763 | 8,74392333 | 0 | 0 |
| path:hsa04917 | Prolactin signaling pathway | 5,615233769 | 160,9709 | 8,331293401 | 0 | 0 |
| path:hsa04218 | Cellular senescence | 5,051045873 | 176,8844456 | 8,285306995 | 0 | 0 |
| path:hsa04114 | Oocyte meiosis | 7,317070987 | 114,4885472 | 8,095141091 | 0 | 0 |
| path:hsa04024 | cAMP signaling pathway | 5,673162352 | 164,252999 | 7,793042563 | 0 | 0 |
| path:hsa04370 | VEGF signaling pathway | 5,491116807 | 85,79468148 | 7,469601017 | 0 | 0 |
| path:hsa00562 | Inositol phosphate metabolism | 6,513603305 | 67,10262946 | 7,406562649 | 0 | 0 |
| path:hsa04913 | Ovarian steroidogenesis | 7,046579272 | 45,01209095 | 7,219906668 | 0 | 0 |
| path:hsa04664 | Fc epsilon RI signaling pathway | 5,321652831 | 75,864209 | 7,034005415 | 0 | 0 |
| path:hsa04666 | Fc gamma R-mediated phagocytosis | 5,493554383 | 74,38638282 | 6,938589179 | 0 | 0 |
| path:hsa04340 | Hedgehog signaling pathway | 6,656947632 | 72,84633834 | 6,933841452 | 0 | 0 |
| path:hsa04020 | Calcium signaling pathway | 5,900958354 | 48,81351263 | 6,743736818 | 0 | 0 |
| path:hsa04360 | Axon guidance | -8,567813338 | 103,9728822 | -6,378179109 | 0 | 0 |
| path:hsa04920 | Adipocytokine signaling pathway | 5,317828571 | 120,5543523 | 6,114021289 | 0 | 0 |
| path:hsa04670 | Leukocyte transendothelial migration | -7,614798295 | 64,80644527 | -6,081857123 | 0 | 0 |
| path:hsa04022 | cGMP-PKG signaling pathway | -7,068031777 | 80,08003082 | -5,663207935 | 0 | 0 |
| path:hsa04928 | Parathyroid hormone synthesis, secretion and action | 3,811941919 | 126,8082061 | 5,578021111 | 0 | 0 |
| path:hsa04621 | NOD-like receptor signaling pathway | -7,23834021 | 145,040538 | -5,495515236 | 0 | 0 |
| path:hsa04650 | Natural killer cell mediated cytotoxicity | 3,094915823 | 81,49229544 | 5,29890327 | 0 | 0 |
| path:hsa04110 | Cell cycle | 3,660329645 | 152,7933055 | 5,240928889 | 0 | 0 |
| path:hsa04510 | Focal adhesion | 1,393912749 | 144,8546582 | 5,10346321 | 0 | 0 |
| path:hsa04921 | Oxytocin signaling pathway | -7,131087618 | 101,6671267 | -5,08012143 | 0 | 0 |
| path:hsa04350 | TGF-beta signaling pathway | -5,688067206 | 134,7072061 | -4,574809342 | 0 | 0 |
| path:hsa04912 | GnRH signaling pathway | 2,499878889 | 134,8040672 | 4,272689886 | 0 | 0 |
| path:hsa04722 | Neurotrophin signaling pathway | 0,989668314 | 172,4353659 | 4,217531153 | 0 | 0 |
| path:hsa04658 | Th1 and Th2 cell differentiation | -5,494035362 | 136,0205813 | -4,164477971 | 0 | 0 |
| path:hsa04726 | Serotonergic synapse | 2,851220308 | 40,8084486 | 4,080838561 | 0 | 0 |
| path:hsa04910 | Insulin signaling pathway | 1,371648031 | 118,0627765 | 3,71438513 | 0 | 0 |
| path:hsa04612 | Antigen processing and presentation | 3,598546774 | 42,57880102 | 3,625658289 | 0 | 0 |
| path:hsa04520 | Adherens junction | -4,914227556 | 117,3158573 | -3,550389218 | 0 | 0 |
| path:hsa04728 | Dopaminergic synapse | 2,661615711 | 80,90481414 | 3,408395312 | 0 | 0 |
| path:hsa00270 | Cysteine and methionine metabolism | -3,436582915 | 46,48497164 | -3,288103047 | 0 | 0 |
| path:hsa04659 | Th17 cell differentiation | 1,73667739 | 145,2721715 | 3,204832073 | 0 | 0 |
| path:hsa04215 | Apoptosis - multiple species | -3,480593695 | 46,00580444 | -3,177956502 | 0 | 0 |
| path:hsa04657 | IL-17 signaling pathway | 2,787144919 | 123,466414 | 3,042926077 | 0 | 0 |
| path:hsa04922 | Glucagon signaling pathway | -3,327603338 | 57,54681471 | -2,919485835 | 0 | 0 |
| path:hsa04625 | C-type lectin receptor signaling pathway | 0,498710344 | 167,4726315 | 2,536269683 | 0 | 0 |
| path:hsa04310 | Wnt signaling pathway | -3,720977227 | 174,970501 | -2,503860946 | 0 | 0 |
| path:hsa04330 | Notch signaling pathway | -2,490189279 | 87,63855738 | -2,185039634 | 0 | 0 |
| path:hsa04060 | Cytokine-cytokine receptor interaction | 1,267291193 | 112,6969358 | 2,145876217 | 0 | 0 |
| path:hsa04730 | Long-term depression | 1,137434188 | 45,430401 | 2,122797769 | 0 | 0 |
| path:hsa04141 | Protein processing in endoplasmic reticulum | 1,886195476 | 54,70120769 | 2,069953995 | 0 | 0 |
| path:hsa04720 | Long-term potentiation | -2,911304483 | 46,61956006 | -2,027856838 | 0 | 0 |
| path:hsa04068 | FoxO signaling pathway | -5,482667177 | 151,9102702 | -1,958153684 | 0 | 0 |
| path:hsa04540 | Gap junction | 0,380812263 | 45,9729722 | 1,934772946 | 0 | 0 |
| path:hsa04144 | Endocytosis | 1,072512074 | 108,3216583 | 1,899940429 | 0 | 0 |
| path:hsa04750 | Inflammatory mediator regulation of TRP channels | 0,851869705 | 58,62048697 | 1,873131251 | 0 | 0 |
| path:hsa04714 | Thermogenesis | -2,682059714 | 69,62715886 | -1,789894211 | 0 | 0 |
| path:hsa04071 | Sphingolipid signaling pathway | -4,215391955 | 169,6188416 | -1,757352014 | 0 | 0 |
| path:hsa04390 | Hippo signaling pathway | -2,911374524 | 163,8735933 | -1,483978157 | 0 | 0 |
| path:hsa04064 | NF-kappa B signaling pathway | 0,262185786 | 123,3842217 | 1,319858015 | 0 | 0 |
| path:hsa04961 | Endocrine and other factor-regulated calcium reabsorption | 0,917086215 | 72,54005521 | 1,273041494 | 0 | 0 |
| path:hsa04261 | Adrenergic signaling in cardiomyocytes | -0,001889092 | 71,06957301 | 1,164096922 | 0 | 0 |
| path:hsa04514 | Cell adhesion molecules (CAMs) | -1,638526135 | 40,30515551 | -1,05790822 | 0 | 0 |
| path:hsa04216 | Ferroptosis | 0,675115021 | 90,32069044 | 0,894824795 | 0 | 0 |
| path:hsa01100 | Metabolic pathways | -2,451064704 | 131,3145036 | 0,854597625 | 0 | 0 |
| path:hsa04371 | Apelin signaling pathway | -1,342497351 | 128,3218226 | 0,844375016 | 0 | 0 |
| path:hsa04061 | Viral protein interaction with cytokine and cytokine receptor | -1,046439294 | 58,72914766 | -0,735149719 | 0 | 0 |
| path:hsa04137 | Mitophagy - animal | -0,360553078 | 132,6293247 | 0,720719511 | 0 | 0 |
| path:hsa04925 | Aldosterone synthesis and secretion | -0,846355451 | 38,89964494 | -0,598856738 | 0 | 0 |
| path:hsa04623 | Cytosolic DNA-sensing pathway | 0,311029484 | 104,9896366 | 0,575589168 | 0 | 0 |
| path:hsa04622 | RIG-I-like receptor signaling pathway | -0,120514561 | 116,7926341 | 0,558532059 | 0 | 0 |
| path:hsa04217 | Necroptosis | -0,326179878 | 102,0338687 | 0,514081741 | 0 | 0 |
| path:hsa04512 | ECM-receptor interaction | -0,774656268 | 47,3335375 | -0,507817399 | 0 | 0 |
| path:hsa00310 | Lysine degradation | 0,320905205 | 45,49584948 | 0,450087743 | 0 | 0 |
| path:hsa04916 | Melanogenesis | -1,540955222 | 83,19349093 | -0,285880124 | 0 | 0 |
| path:hsa04015 | Rap1 signaling pathway | -3,217646565 | 124,5313268 | 0,280642994 | 0 | 0 |
| path:hsa04611 | Platelet activation | -1,632227491 | 95,23159445 | 0,162052851 | 0 | 0 |
| path:hsa04927 | Cortisol synthesis and secretion | -0,494010498 | 62,78701049 | -0,141229637 | 0 | 0 |
| path:hsa04973 | Carbohydrate digestion and absorption | 9,26337534 | 30,39245226 | 9,856636904 | 1,28E-13 | 3,15E-13 |
| path:hsa04270 | Vascular smooth muscle contraction | -8,163605135 | 38,1943697 | -7,265439138 | 1,77E-12 | 4,31E-12 |
| path:hsa00590 | Arachidonic acid metabolism | 6,580420045 | 33,49064979 | 6,770690191 | 4,42E-12 | 1,07E-11 |
| path:hsa04960 | Aldosterone-regulated sodium reabsorption | 7,542327495 | 28,07256595 | 8,253324799 | 2,62E-11 | 6,28E-11 |
| path:hsa00020 | Citrate cycle (TCA cycle) | -4,267184826 | 15,11002609 | -4,240619753 | 1,41E-08 | 3,34E-08 |
| path:hsa04723 | Retrograde endocannabinoid signaling | -3,888689906 | 26,30113567 | -3,124111608 | 2,34E-08 | 5,50E-08 |
| path:hsa01200 | Carbon metabolism | -4,709311457 | 20,10369964 | -4,561766885 | 5,14E-08 | 1,20E-07 |
| path:hsa04976 | Bile secretion | -3,201166132 | 20,11556408 | -3,171535791 | 6,43E-08 | 1,48E-07 |
| path:hsa04918 | Thyroid hormone synthesis | 0,41893106 | 28,46853769 | 0,644480421 | 1,10E-07 | 2,52E-07 |
| path:hsa03460 | Fanconi anemia pathway | 0,488943739 | 26,61821542 | 0,453914717 | 1,13E-07 | 2,56E-07 |
| path:hsa04911 | Insulin secretion | -0,76191248 | 28,2853555 | -0,50852594 | 1,87E-07 | 4,19E-07 |
| path:hsa04713 | Circadian entrainment | -0,338213929 | 30,55797388 | 0,310883763 | 1,89E-07 | 4,21E-07 |
| path:hsa04080 | Neuroactive ligand-receptor interaction | 0,199911551 | 27,47632754 | 0,624974902 | 2,55E-07 | 5,61E-07 |
| path:hsa04710 | Circadian rhythm | 0,067518503 | 27,40799875 | 0,136224673 | 3,00E-07 | 6,56E-07 |
| path:hsa04924 | Renin secretion | 1,817359638 | 22,1315126 | 2,254984244 | 4,31E-07 | 9,32E-07 |
| path:hsa00760 | Nicotinate and nicotinamide metabolism | 0,77464393 | 22,25952164 | 0,865403838 | 5,34E-07 | 1,15E-06 |
| path:hsa04962 | Vasopressin-regulated water reabsorption | 1,064637265 | 19,26198967 | 1,09841294 | 5,97E-07 | 1,27E-06 |
| path:hsa03013 | RNA transport | -0,421619853 | 25,52550349 | -0,266804086 | 6,52E-07 | 1,38E-06 |
| path:hsa03320 | PPAR signaling pathway | -1,789285115 | 20,74187806 | -1,596393732 | 6,88E-07 | 1,42E-06 |
| path:hsa00520 | Amino sugar and nucleotide sugar metabolism | 0,837104589 | 5,923676905 | 0,837348021 | 6,88E-07 | 1,42E-06 |
| path:hsa00524 | Neomycin, kanamycin and gentamicin biosynthesis | 0,72589457 | 5,912530246 | 0,722550958 | 6,88E-07 | 1,42E-06 |
| path:hsa04971 | Gastric acid secretion | -2,677044276 | 18,69608677 | -2,229113869 | 3,14E-06 | 6,40E-06 |
| path:hsa00982 | Drug metabolism - cytochrome P450 | 1,433062331 | 13,82588163 | 1,444947357 | 3,69E-06 | 7,47E-06 |
| path:hsa00790 | Folate biosynthesis | 0,181168582 | 19,73105288 | 0,175782042 | 3,94E-06 | 7,91E-06 |
| path:hsa03015 | mRNA surveillance pathway | -2,289859103 | 14,57940488 | -2,278543517 | 4,76E-06 | 9,46E-06 |
| path:hsa04610 | Complement and coagulation cascades | 1,166932539 | 16,04287161 | 1,170288645 | 4,79E-06 | 9,46E-06 |
| path:hsa00830 | Retinol metabolism | 1,295815568 | 9,372323771 | 1,303391452 | 8,35E-06 | 1,63E-05 |
| path:hsa03440 | Homologous recombination | -0,836393389 | 16,08041335 | -0,808422593 | 8,38E-06 | 1,63E-05 |
| path:hsa00330 | Arginine and proline metabolism | -3,827145805 | 13,64483314 | -3,754164237 | 8,59E-06 | 1,66E-05 |
| path:hsa04136 | Autophagy - other | -0,12835976 | 22,82485294 | 0,203823463 | 1,01E-05 | 1,93E-05 |
| path:hsa00564 | Glycerophospholipid metabolism | 1,585969376 | 13,30880927 | 1,651635375 | 1,11E-05 | 2,11E-05 |
| path:hsa04392 | Hippo signaling pathway - multiple species | 0,153600288 | 19,06330353 | 0,244780466 | 2,08E-05 | 3,92E-05 |
| path:hsa04979 | Cholesterol metabolism | -0,79665362 | 14,99813657 | -0,770899166 | 2,21E-05 | 4,13E-05 |
| path:hsa04672 | Intestinal immune network for IgA production | 2,162461299 | 13,04809231 | 2,352593391 | 2,47E-05 | 4,59E-05 |
| path:hsa00220 | Arginine biosynthesis | -2,856083708 | 9,582466218 | -2,806145574 | 3,35E-05 | 6,17E-05 |
| path:hsa00230 | Purine metabolism | -4,203484396 | 12,34735859 | -4,096120955 | 8,47E-05 | 1,55E-04 |
| path:hsa00350 | Tyrosine metabolism | -1,628002598 | 10,98655126 | -1,586018351 | 1,11E-04 | 2,01E-04 |
| path:hsa04970 | Salivary secretion | -1,278569877 | 14,63862175 | -1,03063087 | 1,14E-04 | 2,05E-04 |
| path:hsa00980 | Metabolism of xenobiotics by cytochrome P450 | 0,366164355 | 13,42820958 | 0,411942911 | 1,19E-04 | 2,14E-04 |
| path:hsa00591 | Linoleic acid metabolism | 0,477844246 | 8,242482635 | 0,494892259 | 1,45E-04 | 2,59E-04 |
| path:hsa00480 | Glutathione metabolism | 0,32537798 | 11,96869331 | 0,345190339 | 1,50E-04 | 2,65E-04 |
| path:hsa00563 | Glycosylphosphatidylinositol (GPI)-anchor biosynthesis | 1,195654864 | 5,82666549 | 1,259513228 | 2,51E-04 | 4,40E-04 |
| path:hsa00140 | Steroid hormone biosynthesis | -1,109691337 | 10,6544011 | -1,041542296 | 3,62E-04 | 6,30E-04 |
| path:hsa04978 | Mineral absorption | 0,580612601 | 12,00974823 | 0,733605949 | 3,84E-04 | 6,64E-04 |
| path:hsa00620 | Pyruvate metabolism | -3,466814982 | 7,514581609 | -3,433252716 | 4,03E-04 | 6,93E-04 |
| path:hsa00380 | Tryptophan metabolism | -2,124423013 | 7,700085338 | -2,086353152 | 4,34E-04 | 7,39E-04 |
| path:hsa04972 | Pancreatic secretion | -0,419922512 | 14,12277648 | -0,15774068 | 4,72E-04 | 8,00E-04 |
| path:hsa00010 | Glycolysis / Gluconeogenesis | -2,215154673 | 8,846136868 | -2,141730824 | 5,45E-04 | 9,16E-04 |
| path:hsa00600 | Sphingolipid metabolism | 2,804163302 | 6,738974375 | 2,83398622 | 6,85E-04 | 0,001143973 |
| path:hsa04724 | Glutamatergic synapse | -1,555192251 | 12,50259547 | -0,922993889 | 7,00E-04 | 0,001162587 |
| path:hsa00250 | Alanine, aspartate and glutamate metabolism | -3,627122073 | 4,280692482 | -3,596182307 | 9,62E-04 | 0,001585869 |
| path:hsa01230 | Biosynthesis of amino acids | -3,896602123 | 7,491950149 | -3,714356037 | 0,001175639 | 0,001925883 |
| path:hsa00260 | Glycine, serine and threonine metabolism | 0,939650221 | 7,089264679 | 0,966554537 | 0,001546588 | 0,002516997 |
| path:hsa01210 | 2-Oxocarboxylic acid metabolism | -0,447149726 | 5,764639832 | -0,444960906 | 0,002263756 | 0,003660228 |
| path:hsa01212 | Fatty acid metabolism | -1,223546935 | 7,101805253 | -1,180258444 | 0,002429858 | 0,00390345 |
| path:hsa00062 | Fatty acid elongation | -1,055742033 | 6,025077579 | -1,044796192 | 0,002565201 | 0,004094456 |
| path:hsa00051 | Fructose and mannose metabolism | 0,860019737 | 5,157076777 | 0,86826671 | 0,003509478 | 0,005565988 |
| path:hsa00730 | Thiamine metabolism | 0,165882855 | 5,691658398 | 0,166464637 | 0,003723118 | 0,005867446 |
| path:hsa04975 | Fat digestion and absorption | 0,320528931 | 4,699647392 | 0,329065973 | 0,0038377 | 0,006009983 |
| path:hsa00500 | Starch and sucrose metabolism | 0,669400708 | 4,453183521 | 0,673174151 | 0,003889218 | 0,006052595 |
| path:hsa04260 | Cardiac muscle contraction | -0,21035109 | 8,887718033 | -0,205739502 | 0,004023404 | 0,006222532 |
| path:hsa00670 | One carbon pool by folate | -0,053757783 | 9,336141409 | -0,047561585 | 0,004985854 | 0,007663442 |
| path:hsa00640 | Propanoate metabolism | -2,36735869 | 4,308827951 | -2,356880552 | 0,005642957 | 0,008620223 |
| path:hsa04977 | Vitamin digestion and absorption | -0,310398953 | 0 | -0,310734758 | 0,005997001 | 0,009105203 |
| path:hsa00052 | Galactose metabolism | 0,755612499 | 4,548817841 | 0,764342437 | 0,006261492 | 0,009449161 |
| path:hsa00983 | Drug metabolism - other enzymes | 0,093104214 | 8,445067453 | 0,15811195 | 0,006317658 | 0,009476487 |
| path:hsa00561 | Glycerolipid metabolism | 0,303415703 | 5,290737357 | 0,334112188 | 0,006671613 | 0,009947495 |
