# Supplementary material for: RNAdetector: a free user-friendly stand-alone and cloud-based system for RNA-Seq data analysis
Source: BMC Bioinformatics. 2021 Jun 3;22:298. doi: 10.1186/s12859-021-04211-7 (PMC8173825; doi:10.1186/s12859-021-04211-7)
Supplement: Supplementary file 4 — Additional file 4. Table with feature comparisons of RNAdetector vs other RNA-Seq pipelines. The table reports the comparison of the features between RNAdetector and the 19 previously published RNA-Seq pipelines. [file 12859_2021_4211_MOESM4_ESM.docx]

|  | **Deployment** | **Supported OS** | **Offline** | **GUI** | **Sequencing input files supported** | **Aligners** | **Gene\transcript quantification and normalization** | **Read count summarization tools** | **Differential expression analysis tools** | **Downstream analysis** | **Settings for ncRNA analysis** | **Multi-species supported** | **Graphical final report** |
| --- | --- | --- | --- | --- | --- | --- | --- | --- | --- | --- | --- | --- | --- |
| **RNAdetector** | Stand-alone or cloud-based. Deployment with Docker. | Windows MacOS  Linux | ✓ | ✓ | FASTQ,  BAM,  SAM | STAR  HISAT2  BWA  SALMON | ✓ | featureCounts  HTSeq  SALMON  CIRI and CIRIquant for circRNAs | DESeq  edgeR  LIMMA | miRNA-sensitive topological pathway analysis | ✓ | ✓ | ✓ |
| **ArrayExpressHTS** | Bioconductor. It can be run locally or remotely at EBI cloud | Linux  MacOS | ✓ | - | FASTQ | BOWTIE TOPHAT  BWA | ✓ | Cufflinks  MMSEQ | - | - | - | ✓ | - |
| **BioJupies** | Web-based application on Jupyter Notebooks. | Windows MacOS  Linux | - | ✓ | FASTQ | Kallisto | ✓ | Kallisto | LIMMA  Characteristic Direction | Several enrichment analyses are supported | - | ✓ | ✓ |
| **BioWardrobe** | Stand-alone  (it seems to be no longer maintained) | MacOS  Linux | ✓ | ✓ | FASTQ  SRA | STAR | ✓ | STAR | DESeq | - | - | ✓ | Only figures |
| **DEWE** | Stand-alone. Deployment with Docker  (it seems to be no longer maintained) | Windows MacOS  Linux | ✓ | ✓ | FASTQ | BOWTIE2 HISAT2 | ✓ | StringTie  HTSeq | Ballgown  edgeR | GSEA | - | ✓ | ✓ |
| **easyRNASeq** | Bioconductor | Windows MacOS  Linux | ✓ | - | BAM | - | ✓ | IRanges  GenomicRanges | - | - | - | ✓ | - |
| **ExpressionPlot** | Standalone software that runs on a virtual machine  (it seems to be no longer maintained) | Windows MacOS  Linux | ✓ | ✓ | FASTQ,  BAM | BOWTIE | ✓ | - | DESeq | - | - | ✓ | ✓ |
| **FX** | Amazon cloud system or it can be installed on local Hadoop clusters  (it seems to be no longer maintained) | Windows MacOS  Linux | - | ✓ | FASTQ  SAM | GSNAP | ✓ | - | - | SNP and INDEL detection | - | Only human and mouse | - |
| **GENE-Counter** | Standalone. Several dependencies are quired. | MacOS  Linux | ✓ | - | FASTQ | CASHX BOWTIE  BWA | ✓ | CASHX | NBPSeq  edgeR  DESeq | GO analysis | - | ✓ | - |
| **GeneProf** | Cloud-based application  (it seems to be no longer maintained) | Windows MacOS  Linux | - | ✓ | SRA importer tool (accession number transferred automatically from  experiment) | BOWTIE TOPHAT | ✓ | - | DESeq  edgeR | GO analysis | - | ✓ | ✓ |
| **Grape RNA-Seq** | Stand-alone. Deployment with Docker or Conda are also available. | MacOS  Linux | ✓ | - | FASTQ  SAM  BAM | GEM | ✓ | FluxCapacitor | - | - | - | ✓ | ✓ |
| **MAP-RSeq** | standalone virtual machine or parallel Sun Grid Engine cluster. Several dependencies are required. | Windows MacOS  Linux | ✓ | - | FASTQ | TOPHAT | ✓ | HTSeq  featureCounts | - | SNP calling,  Fusion transcript detection | - | Only human | ✓ |
| **NGScloud/ NGScloud2** | Cloud application | Windows MacOS  Linux | ✓ | ✓ | FASTQ | BOWTIE2  GSNAP  HISAT2  STAR  TOPHAT | ✓ | Cuffquant  Htseq-count | Cuffdiff  cuffnorm | Variant calling | - | ✓ | - |
| **RAP** | Cloud application | Windows MacOS  Linux | - | ✓ | FASTQ  SRA  BAM  SAM | TOPHAT | ✓ | Cufflinks  HTSeq | Cuffdiff2  DESeq | Splicing junction detection, Exon usage analysis, Fusion transcript detection, Differential polyA analysis | - | ✓ | ✓ |
| **RobiNA** | Stand-alone software | Windows MacOS  Linux | ✓ | ✓ | FASTQ  BAM  SAM | BOWTIE | ✓ | - | DESeq  edgeR | - | - | ✓ | ✓ |
| **RSEQREP** | Stand-alone or cloud-based | Linux | ✓ | - | FASTQ | STAR  HISAT | ✓ | featureCounts | edgeR | Pathway enrichment analysis | - | ✓ | Only figures |
| **RSEQtools** | Stand-alone | MacOS  Linux | ✓ | - | MRF | - | ✓ | mrfQuantifier | - | - | - | ✓ | - |
| **RseqFlow** | Stand-alone tool on Pegasus virtual machine (it seems to be no longer maintained) | Windows MacOS  Linux | ✓ | - | Single ended reads in FASTQ format | BOWTIE PerM | ✓ | - | DESeq | SNP calling | - | ✓ | - |
| **S-MART** | Stand-alone | Windows MacOS  Linux | ✓ | ✓ | FASTQ  SAM | - | ✓ | - | Independent method developed by the authors | - | - | ✓ | Only figures |
| **TCW** | Java desktop application. Several dependencies are required | MacOS  Linux | ✓ | ✓ | FASTA | - | ✓ | - | edgeR  DESeq  EDASeq  DEGseq | GO analysis | - | ✓ | ✓ |
| **TRAPLINE** | Galaxy web application | Windows MacOS  Linux | - | ✓ | FASTQ | TOPHAT2 | ✓ | Cufflinks | Cuffdiff2 | Splicing junction detection, SNP detection, GO analysis, Protein interaction, miRNA target prediction | miRNAs | ✓ | - |
| **wapRNA** | Web application or executable packages for installation on user's local server (it seems to be no longer maintained) | Linux | - | ✓ | FASTA  FASTQ | CoronaLite BWA | ✓ | in-house built Perl module | DEGseq | GO analysis,  KEGG pathway functional enrichment, miRNA target prediction | miRNAs | ✓ | Figures and tables |
