# Supplementary material for: RNAdetector: a free user-friendly stand-alone and cloud-based system for RNA-Seq data analysis
Source: BMC Bioinformatics. 2021 Jun 3;22:298. doi: 10.1186/s12859-021-04211-7 (PMC8173825; doi:10.1186/s12859-021-04211-7)
Supplement: Supplementary file 5 — Additional file 5. Table with feature comparisons of RNAdetector vs other ncRNA-Seq pipelines. The table reports the comparison of the features between RNAdetector and the 7 previously published ncRNA-Seq pipelines. [file 12859_2021_4211_MOESM5_ESM.docx]

|  | **Deployment** | **Supported OS** | **GUI** | **Sequencing input files supported** | **Alignment** | **Counting** | **Differential expression analysis** | **Downstream analysis** | **Regulatory ncRNA supported** | **Multi-organisms supported** | **Graphical final report** |
| --- | --- | --- | --- | --- | --- | --- | --- | --- | --- | --- | --- |
| **RNAdetector** | Docker | Windows  MacOS  Linux | ✓ | FASTQ  BAM  SAM | STAR HISAT2  BWA  SALMON | featureCounts, HTseq, SALMON | DESeq  edgeR  LIMMA | miRNA-sensitive topological pathway analysis (MITHrIL) | miRNAs, snoRNAs, piRNAs, tsRNAs, tUCRs, lncRNAs, circRNAs | ✓ | ✓ |
| **iSmaRT** | Standalone  (website does not work. Not maintained) | Linux | ✓ | FASTQ | BOWTIE | sRNAbench | DESeq  edgeR  NOISeq | GO and pathway enrichment analysis. miRNA\piRNA target prediction. | miRNAs, piRNAs | Human, mouse, rat | Only txt files and figures in output |
| **iSRAP** | Standalone | MacOS  Linux | - | FASTQ  BAM | BOWTIE2 | BEDTools | DESeq  edgeR  LIMMA | - | miRNAs, piRNAs, snoRNAs | ✓ | Only txt files and figures in output |
| **miARma-Seq** | Docker | Windows  MacOS  Linux | - | FASTQ  BAM | BOWTIE2, BWA | featureCounts | edgeR  NOISeq | GO and pathway enrichment analysis, miRNA target prediction | miRNAs, circRNAs | ✓ | Only txt files and figures in output |
| **Oasis 2** | Web-based | Windows  MacOS  Linux | ✓ | FASTQ | STAR | featureCounts | DESeq | GO and pathway enrichment analysis, miRNA target prediction | miRNAs, piRNAs, snoRNAs | ✓ | Only txt files and figures in output |
| **SPORTS1.0** | Standalone | Linux | - | FASTQ | BOWTIE | ? | - | - | miRNAs, piRNAs, snoRNAs, tsRNA | ✓ | Only txt files and figures in output |
| **sRNAnalyzer** | Standalone | MacOS  Linux | - | FASTQ | BOWTIE | ? | - | - | miRNAs, piRNAs, snoRNAs, lncRNA | ✓ | - |
| **sRNApipe** | Galaxy server installed in user’s machine | MacOS  Linux | ✓ | Single-end FASTQ with no adaptors | BWA | ? | - | - | miRNAs, piRNAs, snoRNAs | ✓ | ✓ |
